# Deglycosylation Differentially Regulates Weaned Porcine Gut Alkaline Phosphatase Isoform Functionality along the Longitudinal Axis

**DOI:** 10.3390/pathogens12030407

**Published:** 2023-03-03

**Authors:** Xindi Yin, Weijun Wang, Stephen Y. K. Seah, Yoshinori Mine, Ming Z. Fan

**Affiliations:** 1 Department of Animal Biosciences, University of Guelph, Guelph, ON N1G 2W1, Canada; 2 Key Laboratory of Precision Nutrition and Food Quality, Department of Nutrition and Health, China Agricultural University, Beijing 100083, China; 3 Canadian Food Inspection Agency (CFIA)-Ontario Operation, Guelph, ON N1G 4S9, Canada; 4 Department of Molecular and Cellular Biology, University of Guelph, Guelph, ON N1G 2W1, Canada; 5 Department of Food Science, University of Guelph, Guelph, ON N1G 2W1, Canada; 6 One Health Institute, University of Guelph, Guelph, ON N1G 2W1, Canada

**Keywords:** alkaline phosphatases (AP), deglycosylation and glycosylation, endotoxemia, gut eubiosis and dysbiosis, homeostasis, weaned pig

## Abstract

Gut alkaline phosphatases (AP) dephosphorylate the lipid moiety of endotoxin and other pathogen-associated-molecular patterns members, thus maintaining gut eubiosis and preventing metabolic endotoxemia. Early weaned pigs experience gut dysbiosis, enteric diseases and growth retardation in association with decreased intestinal AP functionality. However, the role of glycosylation in modulation of the weaned porcine gut AP functionality is unclear. Herein three different research approaches were taken to investigate how deglycosylation affected weaned porcine gut AP activity kinetics. In the first approach, weaned porcine jejunal AP isoform (IAP) was fractionated by the fast protein-liquid chromatography and purified IAP fractions were kinetically characterized to be the higher-affinity and lower-capacity glycosylated mature IAP (*p* < 0.05) in comparison with the lower-affinity and higher-capacity non-glycosylated pre-mature IAP. The second approach enzyme activity kinetic analyses showed that *N*-deglycosylation of AP by the peptide *N*-glycosidase-F enzyme reduced (*p* < 0.05) the IAP maximal activity in the jejunum and ileum and decreased AP affinity (*p* < 0.05) in the large intestine. In the third approach, the porcine IAP isoform-X1 (IAPX1) gene was overexpressed in the prokaryotic ClearColiBL21 (DE3) cell and the recombinant porcine IAPX1 was associated with reduced (*p* < 0.05) enzyme affinity and maximal enzyme activity. Therefore, levels of glycosylation can modulate plasticity of weaned porcine gut AP functionality towards maintaining gut microbiome and the whole-body physiological status.

## 1. Introduction

World pork production represents about 35% of the overall meat production, thereby contributing significantly to the world food security, trade and economy [1]. Swine diseases associated with pathogenic bacteria are regarded as the top risk factors that can potentially disrupt normal pork production and pigs are also recognized intermediate hosts for zoonotic diseases [1,2]. Weaned pigs experience gut microbial dysbiosis by harboring zoonotic and opportunistic pathogenic bacteria [3,4], enteric diseases [3], poor efficiency of the digestive utilization of dietary nutrients (i.e., crude protein and dry matter) [4,5] and growth retardation associated with decreased gut alkaline phosphatase (AP) enzyme catalytic capacity and affinity [6]. It is important to further enhance porcine gut AP functionality for optimizing their overall gut environment and function to effectively mitigate the challenges of weaned-pig nutrition, use of feed antimicrobials and the antimicrobial resistance threat arising from pork production activities to the public health [7,8,9].

Gut APs are a group of the most abundant apical membrane-bound glycoproteins in the gut [10]. Gut eubiosis is a status under which gut microbiota are balanced with beneficial bacterial species in mutually maintaining healthy host-microbial relationships in preventing gut dysbiosis [11]. Gut APs play vital eubiotic roles by detoxifying endotoxin lipopolysaccharides (LPS) [7,12], and dephosphorylating other emblematic members of pathogen-associated-molecular patterns (PAMPs) molecules such as triphosphate nucleotides in promoting healthy gut commensal microbiota [8,13,14,15], and attenuating metabolic endotoxemia and syndromes [16,17]. Thus, the gut AP functionality is essential in maintaining gut eubiosis and whole-body normal physiology.

The main determinants of the AP functionality in the gut include the enzymatic maximal activity (*V_max_*) and catalytic affinity (*K_m_*), and these biochemical properties are dependent upon AP enzyme protein structure [6,18]. It has been well documented that concentrations of typical AP physiological substrates, consisting primarily of the PAMPs molecules such as LPS and triphosphate nucleotides (e.g., ATP), are relatively low in the gut luminal environment, e.g., luminal LPS reported at 1.8 µg/mL [19] and extracellular concentrations of triphosphate nucleotides reviewed at 0.4–6 µM [20]. Whereas relatively very large gut AP *K_m_* values (i.e., low AP affinity) have been reported for these physiological substrates in humans (*K_m_* for LPS = 0.35–0.62 mg/mL; and *K_m_* for ATP = 15–32 µM) [21] and pigs (*K_m_* for LPS = 1.35 mg/mL; and *K_m_* for ATP = 1.25 mM) [22] under typical physiological conditions. Taken together, these literature reports strongly support the notion by Millán [23,24] in stating that the AP catalytic affinity is considered limiting to the enzyme functionality.

Mammalian AP structures have been relatively well reported [23,24,25,26,27]. The mammalian multigene AP family isoforms share a similar catalytic site with an active center of a serine residue being surrounded by two zinc ions; these two zinc ions are crucial for enzymatic activity and one magnesium ion is essential for the enzyme stabilization; and when these AP enzymes are activated, their argine-166 residue is essential in substrate binding [23,24,25,26,27]. These mammalian AP family isoforms can still vary in their protein structures, molecular weights as well as other basic biochemical properties such as isoelectric point and kinetic properties [23,24,25,28]. Protein glycosylation, including *N*-linked and *O*-linked glycosylation, can co- or post-translationally further modify various enzyme structures and functionality with the covalent attachment of saccharide moieties to an enzyme protein [29,30]. Associations between enzyme protein glycosylation modifications and various diseases have been established [30].

Albeit of limited reporting of *O*-glycosylation in regulation of AP properties, *N*-glycosylation in modulation of AP expression and functionality has been more widely reported. Through treatments of AP isoenzymes with various exo- and endoglycosidases, it was shown that both *N*- and *O*-glycosylation played essential roles in human tissue-nonspecific AP (TNAP)-associated immunity responses [31]. Glycosylation variation in causing hypophosphatasia in chronic renal and bone diseases resulted from different catalytic properties of TNAP isoforms [32]. A study using site-directed mutagenesis indicated that human TNAP *N*-glycans at specific sites were critical in dimerization that was associated with infantile hypophosphatasia and stable expression of functional TNAP [33]. Bublitz et al. [34] examined roles of *N*-glycosylation in calf enterocytic vesicle-mediated exocytotic export of AP into gut lumen; however, they did not find any link to AP functionality. Through a using *N*-glycosylation inhibitor, López-Posadas et al. [35] demonstrated that enterocytic oxidative stress induced increases in TNAP activity and this was mediated via up-regulating *N*-glycosylation. Forsberg et al. [36] reported the use of the peptide *N*-glycosidase F enzyme in the complete cleavage of *N*-glycosylation for clarification of roles of the *N*-glycosylation in phytase and acid AP functionality of their bacterial phytase expressed in the transgenic phytase pig. Weaning-associated reduction in the intestinal AP digestive capacity could have been partially attributed to the villus enterocytic atrophy and further linked to insufficient enteral nutrient supply; whereas the role of glycosylation in modulation of the AP functionality still needs to be clarified along the intestinal longitudinal axis in the weaned pig [6,37]. Considering pigs are also a relevant large animal model for studying human gastrointestinal physiology, chronic bowel diseases and nutrition [38,39], further understanding how glycosylation regulates AP functionality in the weaned porcine gut will contribute to not only the development of sustainable pork production, but also the enhancement of critical human health management.

Therefore, the primary objective of this study was to take three different research strategies to elucidate the role of glycosylation in regulation of AP isoform functionality in the weanling porcine gut. It is a common belief that *N*- and *O*-glycosylation is limited in bacteria [40]. This was evident from our previous study that a bacterial phytase was not *N*-glycosylated when overexpressed in *E. coli*, whereas this bacterial phytase was heavily *N*-glycosylated when expressed in the transgenic phytase pig [36]. Here we also reported the overexpression of the porcine intestinal AP (IAP) isoform-X1 (IAPX1) in an endotoxin-free *E. coli* strain, referred to as the ClearColiBL21 (DE3) as developed by Mamat et al. [41]. We not only further investigated the porcine intestinal IAPX1 isoform glycosylation and functionality, but also exploited this porcine intestinal IAPX1 isoform’s biochemical properties as a potential exogenous AP in pig nutrition and veterinary pharmaceutical applications.

## 2. Results

### 2.1. Genomic Identification of Multiple Porcine AP Genes and AP Isoforms in the Porcine Gut

To investigate potential differences among different porcine AP isoforms in association with the glycosylation and AP functionality, we have identified three distinct porcine IAP genes located in chromosome 15 and 2 TNAP genes in chromosome 6, representing five AP isoforms from the genome database of *Sus scrofa* (Ver. 11.1) (Table 1). The three identified porcine intestinal-type AP isoforms (IAPX1, accession number XP_003133773.1, link; IAPX2, accession number XP_020930975.1, link; and IAPX3, accession number XP_003133777.1, link) are highly homologous with around 80% identical in their coding amino acid (AA) sequences. Whereas the two porcine TNAP isoforms (TNAPX1, accession number XP_020953340.1, link; TNAPX2, accession number XP_020953343.1, link) are more diverse and are only about 60% identical in their coding AA sequences to the three porcine IAP isoforms. The predicted non-glycosylated and pre-mature coding AA-based molecular weights (MW) for the three porcine IAP isoforms IAPX1, IAPX2 and IAPX3 are the same at about 53 kDa, whereas these predicted MW differed at 61 and 53 kDa, respectively, for the two TNAP isoforms TNAPX1 and TNAPX2 (Table 1).

There is only one predicted *N*-glycosylation site for both IAPX1 and IAPX2 at N141; and there are two predicted *N*-glycosylation sites for IAPX3 at N141 and N421. The predicted *N*-glycosylation sites are comparatively more dynamic in terms of sites and numbers for both TNAPX1 at N279, N320, N352 and N479; and for TNAPX2 at N230, N271, N303 and N430, respectively (Table 1). Thus, there are likely limited differences among the porcine IAP isoforms IAPX1, IAPX2 and IAPX3. However, there are relatively larger differences between the porcine IAP and the TNAP isoforms in terms of the roles of *N*-glycosylation in modulation of these porcine AP isoform functionality. On the other hand, *O*-glycosylation is formed via functional hydroxyl groups with serine, threonine and proline [30]; and accurate prediction of *O*-glycosylation sites is still challenging [42,43]. Thus, we did not pursue the prediction of *O*-glycosylation sites in these target porcine AP isoforms in this study.

### 2.2. Biochemical Characterization of the Porcine AP Isoform Activity along the Porcine Intestinal Longitudinal Axis

**Figure 1:**
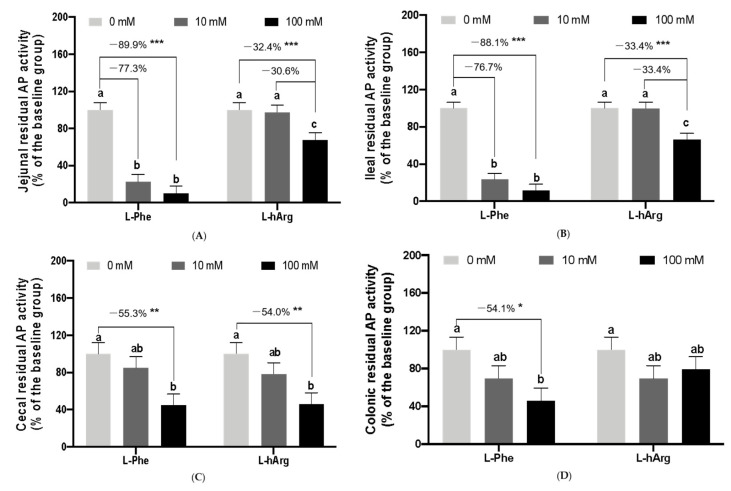
Biochemical identification of alkaline phosphatase (AP) isoforms expressed in (**A**) jejunum (**B**) ileum (**C**) cecum and (**D**) colon of the weanling pigs through conducting inhibition experiments with effects of two specific mammalian AP isoforms’ inhibitors of L-phenylalanine (L-Phe, a specific inhibitor of IAP) and L-homoarginine (L-hArg, a specific inhibitor of TNAP) on the residual AP activity (% of 0-mM inhibitors, i.e., the none-inhibitor baseline groups) at gradient concentrations of 0, 10 and 100 mM, respectively. The residual AP activities (nmol/(mg protein·min)) under the 0-mM of both the inhibitors, i.e., for the none-inhibitor baseline groups, were 30.64 ± 3.31 in the jejunal homogenates; 23.25 ± 1.50 in the ileal homogenates; 5.76 ± 0.71 in the cecal homogenates; and 11.63 ± 1.47 in the colonic homogenates, respectively. Values are means ± pooled SEM (n = 6 pigs) across all the treatment groups with a common control group (0-mM inhibitors), as analyzed by a one-way ANOVA. Comparisons among the baseline 0-mM inhibitor control and each of the treatment groups were conducted by using Tukey’s test, with asterisk(s) indicating *, *p* < 0.05; **, *p* < 0.01; and ***, *p* < 0.001, respectively. ab mean different letters within the same treatment group differ, *p* < 0.05.

Compared to the control group at 0 mM L-Phe or L-hArg, 77–90% of the total weaned porcine jejunal AP activity vs. 77–88% of the total ileal AP activity was inhibited (*p* < 0.01) by L-phenylalanine (L-Phe, a specific inhibitor of IAP) at 10 and 100 mM; whereas 3–32% of the total jejunal AP activity vs. 0–33% of the total ileal AP activity was inhibited (*p* < 0.01) by L-homoarginine (L-hArg, a specific inhibitor of TNAP) at 10 and 100 mM in the concentration-dependent manner (Figure 1). These results would suggest that the IAP isoforms were primarily expressed in the weaned porcine jejunum and ileum.

Furthermore, compared to the control group at 0 mM L-Phe or L-hArg, 15–55% of the total weaned porcine cecal AP activity vs. 30–54% of the total colonic AP activity was inhibited (*p* < 0.05) by L-Phe at 10 and 100 mM; whereas 22–54% of the total cecal AP activity vs. 21–30% of the total colonic AP activity was inhibited (*p* < 0.05) by L-hArg at 10 and 100 mM (Figure 1). These results would indicate that both IAP and TNAP isoforms were significantly expressed in the weaned porcine cecum and colon.

### 2.3. The Approach-1-Glycosylation and the Weaned Porcine Jejunal IAP Functionality Examined by the Fast Protein-Liquid Chromatograph Purification in Combination with Kinetic Analyses

**Figure 2:**
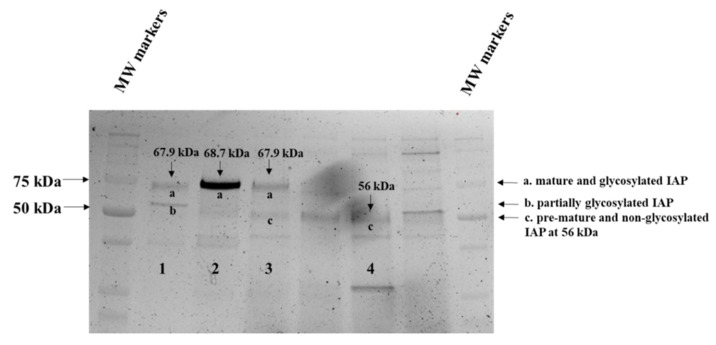
In the Approach-1 research, SDS-PAGE analyses and estimation of molecular weights (MW) of the intestinal-type isoforms of alkaline phosphatase (IAP) fractions that were chromatographically separated and purified from the weanling porcine jejunal tissue homogenates. Lane 1, fraction-1-IAP of glycosylated and partially glycosylated mature IAP mixture; Lane 2, fraction-2-IAP of predominantly glycosylated and mature IAP; Lane 3, fraction-3-IAP of minor glycosylated mature and non-glycosylated pre-mature IAP mixture; and Lane 4, fraction-4-IAP of primarily non-glycosylated pre-mature IAP mixture.

The jejunal homogenate AP protein was solubilized by 1% Triton X-100 and was purified with our established fast protein-liquid chromatograph (FPLC) system by adopting previously reported AP purification procedures [44,45,46,47]. Chromatographically separated and purified IAP protein fractions were further analyzed by the SDS-PAGE and visualized (Figure 2) with their corresponding MW estimated. As visualized in Figure 2, Lane-1-IAP fraction was a mixture of two bands a and b, respectively, representing the highly glycosylated and mature IAP at about 68 kDa and the partially glycosylated IAP; Lane-2-IAP fraction was predominantly the highly glycosylated and mature IAP at about 69 kDa; Lane-3-IAP fraction was a mixture of two bands a and c, respectively, representing the highly glycosylated and mature IAP at about 68 kDa and the non-glycosylated and pre-mature IAP at about 56 kDa; and Lane-4-IAP fraction would primarily represent the non-glycosylated and pre-mature IAP at about 56 kDa. The variations in their estimated MW of these IAP protein fractions might have derived from their different levels of post-translational glycosylation modifications, e.g., distinct glycans’ length and composition.

**Figure 3:**
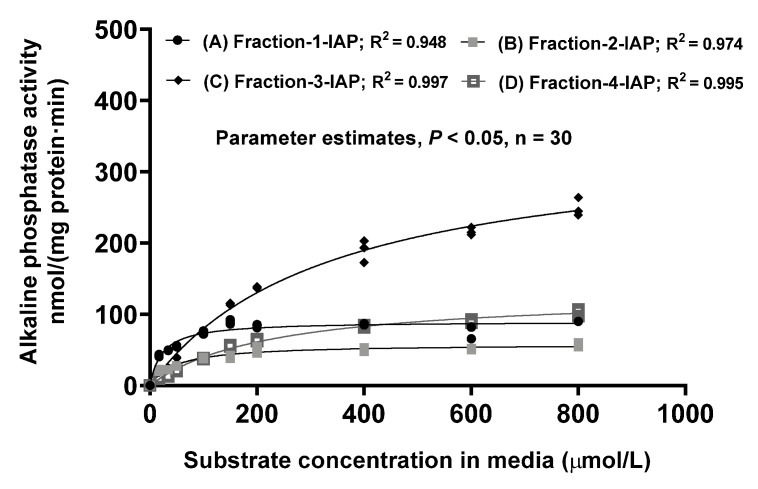
In the Approach-1 research, comparisons of kinetics of activity of hydrolyzing the chromogenic substrate *p*-nitrophenyl phosphate (*p*NPP) by the chromatographically separated and purified porcine intestinal-type isoform alkaline phosphatase (IAP) fractions from the weanling porcine jejunal tissue homogenates. (A) Fraction-1-IAP, glycosylated and partially glycosylated mature IAP mixture; (B) Fraction-2-IAP, predominantly glycosylated and mature IAP; (C) Fraction-3-IAP, minor glycosylated mature and primarily non-glycosylated pre-mature IAP mixture; and (D) Fraction-4-IAP, primarily non-glycosylated pre-mature IAP mixture, as further visualized in Figure 2. Values are means ± SE (n = 4), each kinetic experiment conducted using measurements in 4 replicates at each substrate level and 10 different substrate media concentrations.

Correspondingly, standard AP kinetic analyses were performed on these purified jejunal IAP protein fractions with the chromogenic substrate (*p*NPP) at the physiological pH of 7.4 and 37 °C, as visualized in Figure 3. Kinetics of the chromatographically purified porcine jejunal IAP fractions in hydrolyzing *p*NPP were further compared and summarized in Table 2. Clearly, the Lane-1-IAP and Lane-2-IAP protein fractions, representing the highly glycosylated and mature IAP that were associated with the estimated *K_m_* at 23~53 µM, had averaged 6.4-fold higher (*p* < 0.05) in the IAP enzyme affinity compared with the Lane-3-IAP and the Lane-4-IAP protein fractions, representing the non-glycosylated and pre-mature IAP that were associated with the estimated *K_m_* at 234~327 µM. Furthermore, the Lane-1-IAP and the Lane-2-IAP glycosylated mature IAP protein fractions were associated with the estimated *V_max_* at 57~89 nmol/(mg protein·min) and were thus averaged 2.2-fold lower (*p* < 0.05) in the IAP maximal activity compared with the Lane-3-IAP and the Lane-4-IAP non-glycosylated pre-mature IAP protein fractions that were associated with the estimated *V_max_* at 131~349 µM (Table 2). These results suggest that glycosylation and anchoring on the weaned porcine jejunal apical membrane enabled the mature IAP to be of higher-affinity and lower-capacity whereas the non-glycosylated and pre-mature IAP were characterized to be of lower-affinity and higher-capacity.

### 2.4. The Approach-2-the Role of N-Glycosylation in Modulation of AP Isoform Kinetics along the Weaned Porcine Intestinal Longitudinal Axis

**Figure 4:**
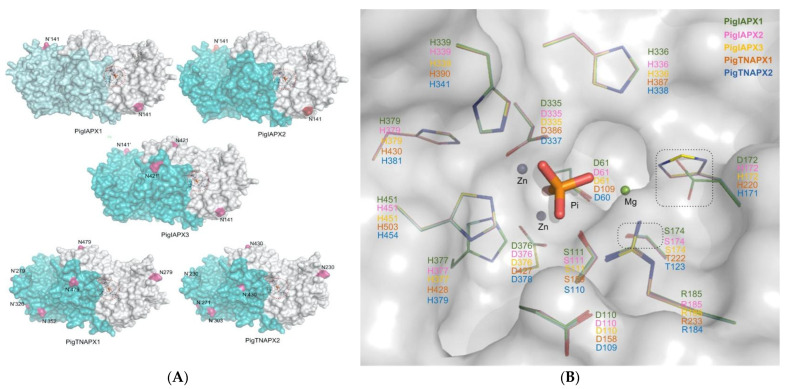
In the Approach-2 research, the 3-D structure homology models mapped with the predicted *N*-glycosylation sites (**A**) and the superimposition of catalytic sites of the five porcine alkaline phosphatase (AP) isoforms (**B**). (**A**) The three-dimensional modeling was generated by the SWISS-MODEL online server using the crystal structure of a homologous human placenta AP (RCSB-PDB#:1EW2) as a template. Structure images were generated using PyMOL (www.pymol.org). One subunit of AP dimer was shown by a cyan surface and another was in white. The predicted *N*-glycosylation sites were colored in pink. (**B**) The five porcine alkaline phosphatase (AP) isoforms, including the intestinal-type AP (IAP) isoform-X1, -X2 and -X3 of IAPX1 (in green), IAPX2 (in pink) and IAPX3 (in yellow) and the tissue-nonspecific alkaline phosphatase (TNAP) isoforms-X1 and -X2 of TNAPX1 (in white) and TNAPX2 (in deep blue). The capital letters represent the short form of amino acid residues.

To further understand the potential role of *N*-glycosylation in the regulation of the functionality of the major weaned porcine IAP isoforms of IAPX1, IAPX2 and IAPX3 and the two TNAP isoforms of TNAPX1 and TNAPX2, the 3-D protein structure models for these five AP isoforms are shown in Figure 4A,B. As revealed through these 3-D protein structure models, although these AP catalytic site architectures, including the heavy metal-binding sites, are highly conserved, the mapped *N*-glycosylation sites are variable among these porcine AP isoforms. The predicted *N*-glycosylation sites for IAPX1 and IAP X2 are at N141; and for IAPX3 are at N141 and N421; for TNAPX1 are at N279, N320, N352 and N479; and for TNAPX2 are at N230, N271, N303 and N430, which are sporadically located within the vicinity of these AP isoforms’ catalytic sites (Figure 4A,B). Thus, differences in the specific number and different *N*-glycosylation sites in these five porcine AP isoforms are likely the major genomic determinants differentially affecting the AP catalytic affinity and maximal enzyme activity along the intestinal longitudinal axis of the weaned pig.

**Figure 5:**
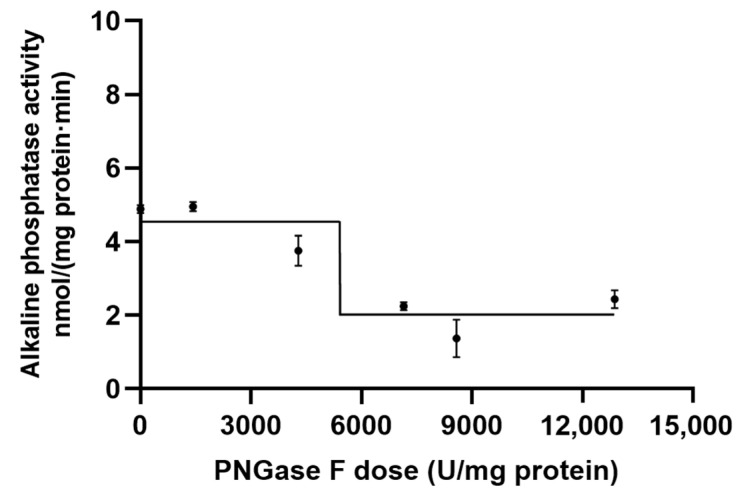
In the Approach-2 research, responses of the alkaline phosphatase (AP) activity (Y, nmol/(mg protein·min) in the weanling porcine jejunal tissue homogenates to gradient treatment doses (x, U/mg jejunal homogenate protein) of the peptide *N*-glycosidase F (PNGase F) enzyme cocktail under in vitro incubations for removal of *N*-glycosylation, as analyzed according to a sigmoidal model (the parameter estimates ± SE) shown in Equation (1) as: Y = 1.98 (±0.26) + [2.97 (±0.01)/(1 + 10 (x/500 − 9.21 (±1.04))], *p* < 0.05 for all the parameter estimates, *R*^2^ = 0.8245, n = 24. The AP activity corresponding to the PNGase-F cocktail doses at the levels of 1, 2, 3, 4, and 6 were, respectively, derived from slopes of the simple linear regression analyses in their time course experiments; and the AP activity at the dose level 5 was derived from the AP kinetics data using the 0.017 mM incubation media substrate of *p*-nitrophenyl phosphate. Values are represented as means ± SE (n = 4).

We then adopted a previous strategy of using the peptide *N*-glycosidase-F enzyme in the complete cleavage of *N*-glycosylation for clarification of roles of the *N*-glycosylation in modulation of phytase and acid AP functionality in the transgenic phytase pig [36]. We conducted a dose-response experiment with the weaned porcine jejunal homogenates and observed sigmoidal responses in the residual AP activity with gradient treatment doses (U/mg jejunal homogenate protein) of the peptide *N*-glycosidase F (PNGase-F) enzyme cocktail under the in vitro incubations of pH of 7.4 at 37 °C, clearly defining the optimal doses of the PNGase-F enzyme cocktail required for the complete cleavage of *N*-glycosylation in vitro treatments in this study (Figure 5).

**Figure 6:**
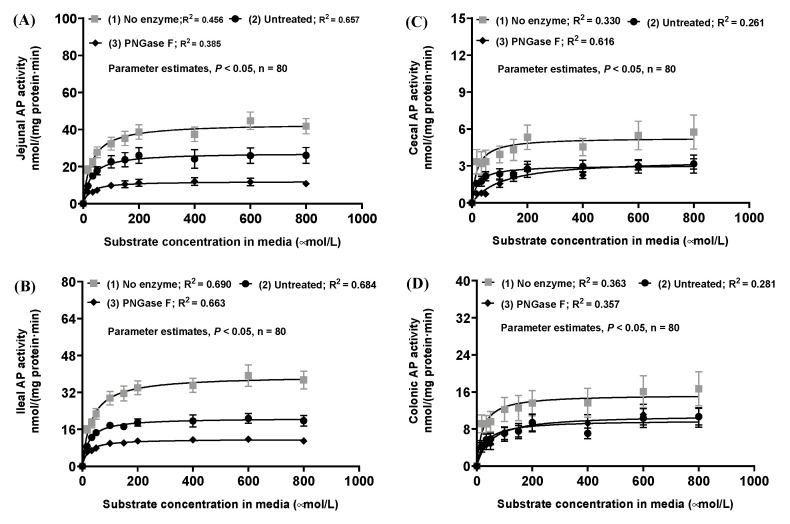
In the Approach-2 research, effects of removal of *N*-glycosylation through using in vitro incubations with the peptide *N*-glycosidase F (PNGase-F) cocktail on the respective gut tissue homogenate alkaline phosphatases (AP) activity kinetics in hydrolyzing the chromogenic substrate P-nitrophenyl phosphate in the AP kinetic experiments after an incubation in the base buffer alone without the PNGase-F enzyme cocktail but subjecting to the intrinsic effects of the respective gut tissue homogenates (as No enzyme group); after an incubation in the base buffer without an inhibitor (as Untreated group); and after an incubation in the incubation mixture containing 7150 U PNGase-F/mg protein of respective gut tissue homogenates (as PNGase-F group) along the intestinal longitudinal axis of (**A**) jejunum, (**B**) ileum, (**C**) cecum and (**D**) colon in the weanling pigs. Values are means ± SE (n = 8), presenting eight independent kinetic experiments conducted by using eight respective weanling porcine gut tissue sample homogenates, each kinetic experiment measured at 10 different substrate concentrations with four replicates at each substrate medial concentration.

We subsequently carried out the in vitro incubations with the respective weaned gut tissue homogenates, containing AP primarily bound in the apical membrane vesicles, with the PNGase-F cocktail at 7150 U PNGase F/mg protein tissue homogenates of jejunum, ileum, cecum and colon in the weaned pigs. Standard AP kinetic analyses were performed on these PNGase-F treated porcine gut tissue homogenate AP, primarily bound in the apical membrane vesicles, with the chromogenic substrate *p*NPP at the physiological pH of 7.4 and 37 °C, as visualized in Figure 6.

Effects of removal of the *N*-glycosylation through in vitro incubations with the PNGase-F cocktail on the respective weaned porcine gut tissue homogenate AP kinetics in hydrolyzing the chromogenic substrate *p*NPP were compared and summarized in Table 3. The PNGase-F treatment reduced (*p* < 0.01) the jejunal and ileal IAP isoform maximal activity by 45~57%; whereas this cleavage of *N*-glycosylation treatment did not affect (*p* > 0.05) both the jejunal and ileal IAP isoform affinity. Furthermore, the PNGase-F treatment tended to decrease (*p* = 0.0787) the cecal AP affinity by 6.3-fold; and decreased (*p* < 0.01) the colonic AP affinity by 51%. However, this cleavage of *N*-glycosylation treatment did not affect (*p* > 0.05) both the cecal and colonical AP maximal activity (Table 3). Interestingly, incubating the jejunal and ileal tissue homogenates alone, without the presence of the PNGase-F the cocktail, resulted in increases (*p* < 0.01) in the jejunal and ileal IAP isoform maximal activity, suggesting that the jejunal and ileal intracellular endogenous lysosomal enzymes could have also played a role in affecting the ileal IAP isoform maximal activity. These results collectively suggest that *N*-glycosylation was important to maintain a higher jejunal and ileal IAP isoform maximal activity without influencing the small intestinal AP isoform enzyme affinity; whereas *N*-glycosylation played a significant role in maintaining the large intestinal AP affinity, likely via specifically upholding the TNAP isoform enzyme affinity in the weaned pig.

### 2.5. The Approach-3-Kinetic Characterizing the Impact of Lack of Glycosylation and IAP Functionality in the Recombinant Porcine IAPX1 Isoform Overexpressed in the ClearColiBL21 (DE3)

To further investigate the impact of lack of glycosylation, including both *N*-linked and *O*-linked glycosylation in porcine IAP functionality, we then overexpressed the porcine IAPX1 in the endotoxin-free ClearColiBL21 (DE3) *E. coli* strain; investigated the glycosylation-deficient IAPX1-function relationship and further exploited functional properties; thus, feasibility of this recombinant porcine IAPX1 isoform as an exogenous porcine IAP for potential pig nutrition and veterinary pharmaceutical applications.

**Figure 7:**
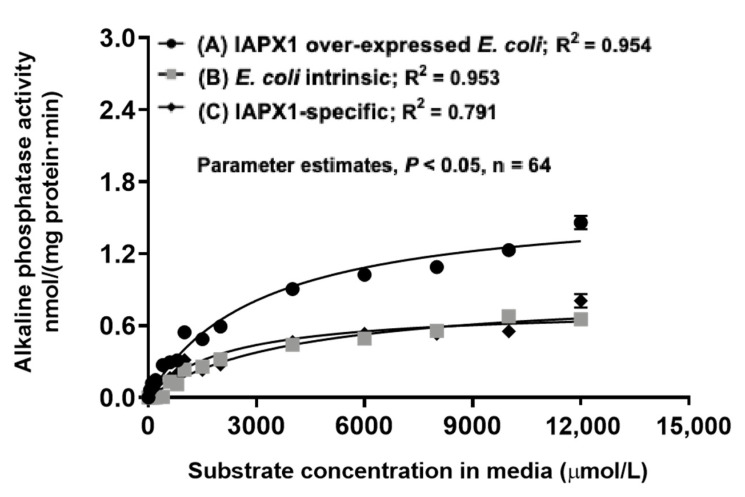
In the Approach-3 research, partitioning of the activity kinetics of the porcine intestinal-type alkaline phosphatase (IAP) isoform-X1 (IAPX1) overexpressed in the ClearColiBL21 (DE3) cell lysates in hydrolyzing the chromogenic substrate *p*-nitrophenyl phosphate. (A) The kinetics of the total AP activity determined with the ClearColiBL21 (DE3) *E. coli* cell lysates processed from the same condition-cultured cells harboring the p15TV-L-IAPX1 and overexpressing IAPX1; (B) the kinetics of the AP activity intrinsic to the *E. coli* (ClearColiBL21 (DE3)) cell lysates processed from the same condition-cultured cells but only harboring the vector p15TV-L alone; and (C) the IAPX1-specific kinetics of the AP activity partitioned between the total AP activity determined with the ClearColiBL21 (DE3) cell lysates overexpressing the IAPX1 and the AP activity only intrinsic to the *E. coli* (ClearColiBL21 (DE3)) cell lysates processed from the same condition-cultured cells but only harboring the vector p15TV-L alone. Values are means ± SE (n = 4), indicating one respective kinetic experiment measured at 16 different substrate media concentrations with four replicates at each substrate concentration.

The recombinant porcine IAPX1 could not be purified via the N-terminal His-tag and the Ni-NTA resin-based affinity chromatography. It was also anticipated that there would be potential technique challenges of trying to separate the bacterial intrinsic AP associated this *E. coli* strain from the recombinant IAPX1 in the ClearColiBL21 (DE3) *E. coli* cell lysate by using the FPLC strategy. The additional consideration and rationale for characterizing this recombinant IAPX1 in the ClearColiBL21 (DE3) *E. coli* cell lysate is that its expenses would be relatively low for the recombinant IAPX1 in its dried crude lysate form in comparison with the dried and purified recombinant IAPX1 for practical field application as an exogenous recombinant porcine AP feed additive once we have proven its in vivo application efficacy and obtained government’s regulatory approval. Thus, with these considerations, we further conducted the IAPX1-specific AP kinetic partitioning with the chromogenic substrate *p*NPP at the physiological pH of 7.4 and 37 °C, as visualized in Figure 7.

The total AP kinetics and the AP activity specific to this *E. coli* strain were determined using cell lysates of ClearColiBL21 (DE3) derived from two types of cultured cells: those harboring p15TV-L-IAPX1 and those harboring the vector p15TV-L alone (Figure 7). Kinetics of AP activity intrinsic to the ClearColiBL21 (DE3) cell lysate were obtained, including *V_max_* of 0.89 ± 0.05 nmol mg protein^−1^·min^−1^ and *K_m_* of 4068.0 ± 525.0 µM; and kinetics of the IAPX1-specific activity were further mathematically partitioned, including *V_max_* of 0.75 ± 0.05 nmol mg protein^−1^·min^−1^ and *K_m_* of 2102.0 ± 450.8 µM (Table 4).

As summarized in Table 4, the recombinant IAPX1-specific enzyme affinity was higher (2102.0 vs. 4068.0 µM, *p* < 0.05) by 94% than the AP activity intrinsic to the *E. coli* (ClearColiBL21 (DE3)). However, the recombinant IAPX1-specific enzyme affinity was 6.5-fold lower (*p* < 0.05) than that of the FPLC purified weaned porcine jejunal non-glycosylated and pre-mature IAP with *K_m_* averaged at 281 µM (Table 4). Because the AP kinetics for both the ClearColiBL21 (DE3) AP and the recombinant IAPX1 were not conducted with their respectively purified enzyme preparations, direct comparison of their *V_max_* would be of limited reference values. Nevertheless, there was no difference (0.75 vs. 0.89 nmol mg protein^−1^·min^−1^, *p* > 0.05) in the maximal enzyme activity *V_max_* between the recombinant IAPX1-specific enzyme and the AP activity intrinsic to the ClearColiBL21 (DE3).

**Figure 8:**
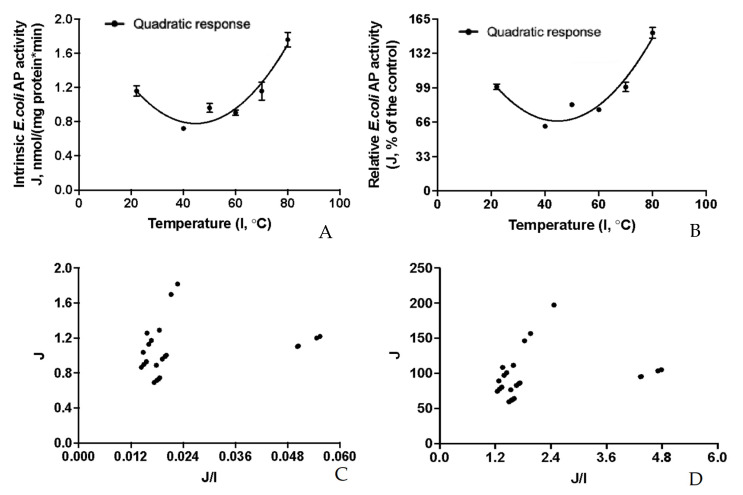
In the Approach-3 research, external thermal treatment effects on the residual alkaline phosphatase (AP) activity intrinsic to the *E. coli* (ClearColiBL21 (DE3)) cell lysates processed from the same condition-cultured cells only harboring the p15TV-L measured by using the chromogenic substrate *p*-nitrophenyl phosphate (*p*NPP) when exposed to the external thermal treatments for 30 min, ranging from 22 to 80 °C. Values are represented as parameter estimates. (**A**) Plot of the inhibition kinetic relationship between the residual *E. coli* AP activity (J, nmol/(mg protein·min)) intrinsic to the *E. coli* (ClearColiBL21 (DE3)) cell lysates against the increasing gradient of external thermal treatments (I, °C) according to the quadratic response model established as: Y = 2.209 (± 0.169) − 0.064 (±0.007)x + 0.0007 (±0.00007)x^2^, *p* < 0.0001 for all the model parameter estimates, *R*^2^ = 0.861, n = 24; and according to the exponential response model shown in Equation (2) established as: Y = 0.5859 (±0.007)e0.0114 (±0.0033)x, *p* < 0.0001 for J_0_, the initial residual *E. coli* AP activity estimate, *p* = 0.0022 for b, the rate constant estimate, *R*^2^ = 0.323, n = 24; (**B**) the plot of the inhibition kinetic relationship between the relative residual *E coli* AP activity (J, % of the positive control group measured at the room temperature at 22 °C intrinsic to the *E. coli* (ClearColiBL21 (DE3)) cell lysates against the increasing gradient of external thermal treatments (I, °C) according to the quadratic response model established as: Y = 190.71 (±14.55) − 5.49 (±0.63)x + 0.061 (±0.006)x^2^, *p* < 0.0001 for all the model parameter estimates, *R*^2^ = 0.861, n = 24; and according to the exponential response model shown in Equation (2) established as: Y = 50.57 (±10.58)e0.0114 (±0.0033)x, *p* < 0.0001 for J_0_, the initial relative residual *E. coli* AP activity estimate, *p* = 0.0022 for b, the rate constant estimate, *R*^2^ = 0.323, n = 24; (**C**) the Eadie–Hofstee linear plot between the residual *E. coli* AP activity (J) and the J/I ratio as: y = 0.920 (±0.122) + 5.444 (±4.399)x, *p* < 0.0001 for the intercept, *p* = 0.2320 for the slope, *r*^2^ = 0.071, n = 24; and (**D**) the Eadie–Hofstee linear plot between the relative residual *E. coli* AP activity (J, % of the positive control group measured at the room temperature at 22 °C) and the J/I ratio as: y = 79.42 (±10.51) + 5.44 (±4.40)x, *p* < 0.0001 for the intercept, *p* = 0.2320 for the slope, *r*^2^ = 0.071, n = 24.

The inhibition kinetic relationships between the residual AP activity and relative AP activity (J) intrinsic to the *E. coli* (ClearColiBL21 (DE3)) cell lysate and the increasing gradient of external thermal treatments (I) were visualized in Figure 8A,B. The thermostability response curves obtained for the intrinsic *E. coli* (ClearColiBL21 (DE3)) were significantly fitted (*p* < 0.05) by a quadratic (shown in the plot) and an exponential curve (not shown in the plot); and the quadratic pattern was much better fitted than the exponential pattern as judged by the *R*^2^ values (*R*^2^ = 0.86 vs. 0.32). To our surprise, the intrinsic *E. coli* residual AP activity and relative AP activity showed curve-linear patterns of increases after 30 min of incubations starting from 22 to 80 °C; and the intrinsic *E. coli* activity was enhanced by about 60% at 80 °C (Figure 8B). However, there were not significant Eadie–Hofstee linear relationships (*p* > 0.05 for the linear term slopes) between the intrinsic *E. coli* AP activities (J) and the J/I ratios (Figure 8C,D), further suggesting these thermal treatment inhibition kinetic parameter estimates could not be obtained from these Eadie–Hofstee analyses (Table 5). Rather than exhibiting inhibitory effects, the thermal treatments of the *E. coli* (ClearColiBL21 (DE3)) cell lysate from 22 to 80 °C for 30 min could actually curve-linearly increase the intrinsic *E. coli* AP activity.

**Figure 9:**
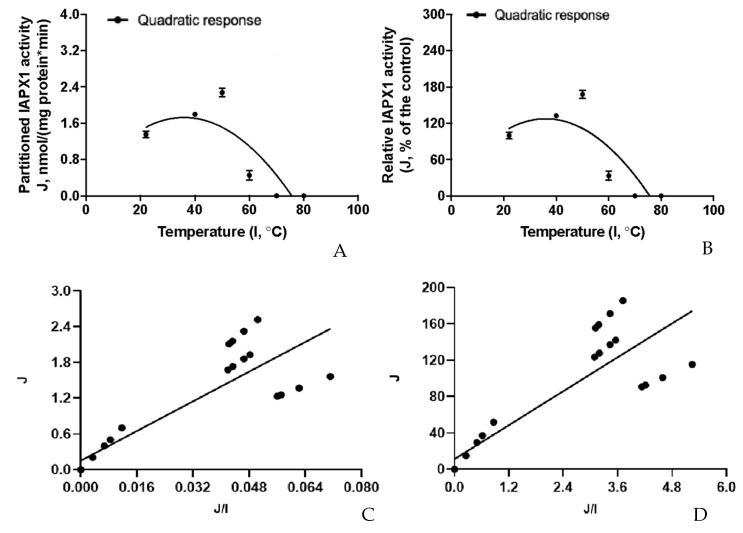
In the Approach-3 research, external thermal treatment effects on the residual porcine intestinal-type alkaline phosphatase (IAP) isoform-X1 (IAPX1)-specific activity partitioned between the total AP activity determined with the ClearColiBL21 (DE3) cell lysates overexpressing IAPX1 and the AP activity only intrinsic to the *E. coli* (ClearColiBL21 (DE3)) cell lysates measured by using the chromogenic substrate *p*-nitrophenyl phosphate (*p*NPP) when exposed to the external thermal treatments for 30 min, ranging from 22 to 80 °C. Values are represented as parameter estimates. (**A**) the quadratic response model established as: Y = 0.3034 (±0.7861) + 0.0792 (±0.0324)x − 0.0011 (±0.0003)x^2^, *p* = 0.6968 for the intercept estimate, *p* = 0.0234 for the linear slope estimate, *p* = 0.0020 for the quadratic slope estimate, *R*^2^ = 0.690, n = 24; and according to the exponential response model shown in Equation (2) established as: Y = 3.014 (±0.982)e − 0.0218 (±0.0078)x, *p* = 0.0056 for J_0_, the initial residual IAPX1-specific AP activity estimate, *p* = 0.0105 for b, the rate constant estimate, *R*^2^ = 0.360, n = 24; (**B**) the plot of inhibition kinetic relationship between the relative residual IAPX1-specific AP activity (J, % of the positive control group measured at the room temperature at 22 °C) against the increasing gradient of external thermal treatments (I, °C) according the quadratic response model established as: Y = 22.38 (±56.66) + 5.84 (±2.39)x − 0.081 (±0.023)x^2^, *p* = 0.6969 for the intercept estimate, *p* = 0.0234 for the linear slope estimate, *p* = 0.0020 for the quadratic slope estimate, *R*^2^ = 0.690, n = 24; and according to the exponential response model shown in Equation (2) established as: Y = 222.30 (±72.46)e − 0.0218 (±0.0078)x, *p* = 0.0056 for J_0_, the initial relative residual IAPXI-specific AP activity estimate, *p* = 0.0105 for b, the rate constant estimate, *R*^2^ = 0.357, n = 24; (**C**) the Eadie–Hofstee linear plot between the residual IAPXI-specific activity (J) and the J/I ratios as: y = 0.1526 (±0.1354) + 31.12 (±3.70)x, *p* = 0.2718 for the intercept, *p* < 0.0001 for the slope, *r*^2^ = 0.762, n = 24; and (**D**) the Eadie–Hofstee linear plot between the relative residual IAPX1-specific activity (J) and the J/I ratios as: y = 11.27 (±9.99) + 31.11 (±3.71)x, *p* = 0.2715 for the intercept, *p* < 0.0001 for the slope, *r*^2^ = 0.762, n = 24.

The inhibition kinetic relationships between the partitioned recombinant porcine IAPX1-specific AP activity overexpressed in the ClearColiBL21 (DE3) cell lysate in responses to the increasing gradient of external thermal treatments were visualized in Figure 9A,B. The thermostability response curves obtained for the partitioned recombinant porcine IAPX1-specific AP activities were significantly fitted (*p* < 0.05) by a quadratic (shown in the plot) and an exponential curve (not shown in the plot); and the quadratic pattern was much better fitted than the exponential pattern as assessed by the *R*^2^ values (*R*^2^ = 0.69 vs. 0.36). There were significant Eadie–Hofstee linear relationships (*p* < 0.05 for the linear term slopes) between partitioned recombinant porcine IAPX1-specific AP activities (J) and the J/I ratios (Figure 9C,D). Thermal treatment inhibition kinetic parameter estimates were obtained from these Eadie–Hofstee analyses for the partitioned recombinant porcine IAPX1-specific AP activity overexpressed in the ClearColiBL21 (DE3) cell lysate (Table 5). The *TC*_50_ was estimated at 31.1 ± 3.71 °C, indicating that the recombinant IAPX1-specific AP would lose 50% its activity after 30 min thermal treatment at 31 °C; whereas it would lose 100% its activity after 30 min thermal treatment at 62 °C. Thus, this porcine recombinant IAPX1 overexpressed in the ClearColiBL21 (DE3) cell was not thermal tolerant.

## 3. Discussion

Glycosylation is a vital post-translational modification for the structure and functional properties of glycoproteins [48]. Glycans are shown to play important and direct roles in substrate recognition, specificity, affinity, turnover rates of enzymes and indirectly via intestinal epithelial interactions with commensal bacteria in maintaining homeostasis and pathogenesis [49,50,51,52]. Gut APs are a critical defense factor of gut epithelia of weaned pigs, and AP activity and expression are affected by nutritional and genomic factors. For example, dietary components such as energy intake, fat content and type, AA, peptides and oligosaccharides have been reviewed to likely regulate IAP expression and activity [53]. Our primary objective was to elucidate the role of glycosylation in control of AP isoform functionality in the weaned porcine gut. Multiple AP isoforms are typically expressed and may be differentially affected by glycosylation in the mammalian gut [24]; however, little is known about AP isoform expression in the porcine gut. Through analyzing porcine genome database, we firstly identified three porcine IAP isoforms IAPX1, IAPX2 and IAPX3 and two TNAP isoforms TNAP1 and TNAP2 that were likely expressed along the porcine gut longitudinal axis. Our previous porcine gut AP kinetic analyses suggested that different AP isoforms would be expressed along the small intestinal longitudinal axis [54]. L-Phe and L-hArg have been established to be the respective mammalian IAP and the TNAP-specific inhibitors in previous human and rodent studies [21,37,55]. We further increased the concentrations of these two inhibitors L-Phe and L-hArg up to 100 mM in the AP isoenzyme inhibition experiments in this study. It should be noted that the small intestinal tissues are multi-cell lineage based, and apart from enterocytes, other cell types such as intraepithelial lymphocytes, vascular endothelia and smooth muscle cells do exit and express the TNAP isoforms, thus helping to explain why L-hArg could inhibit some weaned porcine small intestinal AP activity in these inhibition experiments. Nevertheless, these L-Phe and L-hArg inhibition experimental results collectively suggest that the IAP isoforms were primarily expressed in the weaned porcine jejunum and ileum whereas both IAP and TNAP isoforms were expressed in the weaned porcine cecum and colon. Future research should be conducted to further reveal molecular mechanisms and examine how dietary factors regulate both IAP and TNAP isoforms’ expression along the small—large intestinal longitudinal axis in the weaned pig.

It is well established that intestinal APs are highly glycosylated while being trafficked through intracellular sub-organelles and are anchored on the apical membrane via the GPI bonding as the mature form of AP [46]. Partitioning analysis revealed that the majority (~98%) of the gut mucosal APs was apical membrane-bound, while a minor fraction of intracellular soluble APs was present, likely representing premature APs in weaning pigs [6]. We reasoned that glycosylation associated with the mature APs not only changed the AP MW, but also the AP enzyme molecule ionization and affinity; thus, the highly glycosylated mature APs and the pre-mature APs could be readily separated by FPLC. Whereas the three different porcine IAP isoforms may not be easily further separated by the FPLC procedure because of their highly conserved coding AA sequences and the *N*-glycosylation sites. Jejunum and ileum account for the major small intestinal longitudinal length and have been characterized to primarily express the IAP isoforms in the weaned pig as characterized from the inhibitor experiments of this study. We hence took the FPLC protein purification and enzyme activity kinetic analysis strategy and further investigated the role of glycosylation, including *N*- and *O*-glycosylation, in modulation of the IAP isoform functionality in the weaned porcine small intestine.

The weaned porcine jejunal mature IAP was purified and separated from its pre-mature IAP by the FPLC procedure. The gel band-a shown in the Figure 2 represented the highly mature IAP at about 69 kDa, which is consistent with our previously reported weaning porcine jejunal AP MW at about 60 kD via the Western blotting analyses [6]. The gel band-c shown in the Figure 2 represented the pre-mature IAP MW at about 56 kDa, which is consistent with the coding AA-based predicting of MW for the porcine IAP isoforms at about 53 kDa. Forsberg et al. [36]. reported that an extra 7.6 kDa glycan was added to phytase in the transgenic phytase pigs primarily due to the *N*-glycosylation. The discrepancy in MW at about 13 kDa between the mature and the pre-mature porcine jejunal IAP was presumably due to the combined *N*- and *O*-glycosylation modifications. Thus, our results support the notion that the apical membrane-bound and mature jejunal IAP is highly glycosylated whereas the intracellular pre-mature jejunal IAP is non-glycosylated in the weaned pig.

Biochemical properties of the isolated and purified weaned porcine jejunal mature and glycosylated IAP were further compared with their corresponding pre-mature and non-glycosylated IAP through standard AP kinetic analyses by using the chromogenic substrate *p*NPP at the physiological pH of 7.4 and 37 °C. It is well established that AP kinetics are affected AP assay media pH [21,23,24]. Kinetics of these chromatographically purified porcine jejunal IAP fractions in hydrolyzing *p*NPP in this study were measured at the physiological pH of 7.4. Thus, these IAP kinetic estimates obtained in this study cannot be meaningfully compared with our previously reported porcine gut AP kinetics that were measured at the AP optimal pH at 10.5 [6,21]. The *K_m_* estimated for the mature and highly glycosylated jejunal IAP at 23~53 µM is similar to the *K_m_* of 25.47 ± 4.75 µM for the jejunal mucosal homogenate within this study, and these comparable Km results are consistent with the previous observation that majority (>98%) of the porcine intestinal mucosal AP activity was associated with the membrane-bound mature AP [16]. The mature and highly glycosylated weaned porcine jejunal IAP was estimated to have a *K_m_* at 23~53 µM from this study and is similar to the *K_m_* of 24 ± 6.5 µM measured for the recombinant human IAP measured by using the chromogenic substrate *p*NPP at the physiological pH of 7.4 and 37 °C [21]. These results of this study suggest that glycosylation and anchoring on the epithelial apical membrane enabled the mature jejunal IAP to be of higher-affinity and lower-capacity whereas the intracellular non-glycosylated and pre-mature IAP were characterized to be of lower-affinity and higher-capacity in the weaned pig. Thus, majority of the weaned porcine gut IAP is apical membrane-bound, mature and high-affinity for playing physiological roles.

Under this context, it should be pointed out that intestinal glycosylation patterns, as mediated directly or indirectly via affecting IAP glycosylation, can be influenced by various factors such as diet, microbiota and physiological conditions. The glycans of glycoproteins are located at the interface in the gut and mediate host-microbial interactions and offer microbial binding sites [35]. Most intestinal mucosal digestive enzymes, including IAP on the apical membrane, are glycoproteins. The fucosylation of these enzymes alters due to nutritional manipulation after weaning [56]. Fucosylation can occur directly via *O*-glycosylation or indirectly via *N*-glycosylation via beta-D-galactose [52]. Moreover, it was shown that gut bacteria possess either the glycolytic activity or the capacity to induce glycosylation [57]. Goto et al. [52] showed that the gut health environment and homeostasis such as susceptibility to infection by *Salmonella typhimurium* could be regulated through aberrant intestinal fucosylation, which could have been partly mediated through affecting the host small intestinal IAP functionality.

On other hand, *N*-acetyl-ß-glucosamine is the invariant pentasaccharide core region of the asparagine-linked *N*-glycosylation of the mammalian APs [58]. *N*-Acetyl-ß-glucosamine is the basic monomeric constituent of chitin, chitosan and chito-oligosaccharides that have been well documented to attenuate gut dysbiosis, modulate immunity, growth and metabolic status, thus being regarded as effective prebiotics [59,60,61]. The role of gut APs in detoxifying LPS and dephosphorylating other PAMPs, including triphosphate nucleotides, is well established in promoting a healthy gut microbiota and reducing the risk of metabolic syndromes [7,8,12,13,14,15,16,17]. It is conceivable that the chitin- and chitosan-based prebiotic effects are likely mediated through modulation of the gut endogenous AP functionality via affecting their *N*-glycosylation. Thus, glycosylation, including *N*- and *O*-glycosylation, may modulate porcine small intestinal IAP functional maturation, gut eubiosis and whole-body normal physiological status. It should be pointed out that our *N*-glycosylation impact results in this study were of in vitro nature and the weaned pigs were fed a typical corn and SBM-based commercial diet with a very limited dietary source of *N*-acetyl-ß-glucosamine. Further in vivo studies need to be carried out to further investigate roles of *N*-glycosylation, such as dietary supplementation of exogenous glycans that are rich in *N*-acetyl-ß-glucosamine and/or other hemicellulose sugars, in modulation of both IAP and TNAP isoform expression and functionality as well as microbiota and microbiome responses in the gut of the early weaned pig.

The *N*- and *O*-glycans of glycoproteins are participated in protein folding, trafficking, function and stability [32,33]. The *N*- and *O*-glycan chains of AP isoforms are synthesized with various glycosidases and glycosyltransferases by adding monosaccharides in the path from the endoplasmic reticulum (ER) and Golgi apparatus to the apical membrane anchored via glycosylphosphatidylinositol (GPI) [30,59,62]. It was also shown that *N*-glycan was not a prerequisite for the activity of the placental AP and IAP based on glycosidase digestion in human [31]. Interestingly, it was demonstrated that TNAP lost the catalytic activity upon the removal of *N*-glycan, indicating that *N*-glycan played an essential role in the catalytic activity of TNAP but not in tissue-specific AP [31]. Furthermore, the TNAP isoforms prepared from osteosarcoma showed different catalytic properties as a result of a structural difference in *N*-glycan [32]. It was shown that the oligosaccharide sidechains of the IAP were not ended by sialic acid compared to the other AP isoforms [63]. The sugar chain of purified AP from adult human intestine contained blood group substance but not in fetal intestine [64]. The variety in glycosylation patterns of liver, bone and kidney TNAP isoforms lead to slight differences in electrophoretic mobility and thermos-stability [65,66]. The bone AP isoforms (B1, B2, and B/I) differed in *N*-linked glycans, which resulted in significantly different catalytic properties [32]. A study using site-directed mutagenesis shown that the dimeric structure required for TNAP activity remained in individual single *N*-glycan deletion mutants [33]. The infantile hypophosphatasia was linked to TNAP mutants (N430S) that were glycosylation-defective and unable to dimerize; and the *N*-glycans on N230, N271 and N303 were the minimal requirement for the structure, function and stable expression of TNAP in a cell [33]. Thus, different glycosylation sites may impact the AP enzyme structure and functionality in many ways and different AP isoforms may respond to *N*- or *O*-glycosylation differentially.

Under this context, through analyzing the porcine genome database, we identified the specific *N*-glycosylation sites in the three porcine IAP isoforms IAPX1, IAPX2 and IAPX3 and the two TNAP isoforms TNAPX1 and TNAPX1. We also modelled the 3-D structure homology mapped with the predicted *N*-glycosylation sites and the superimposition of catalytic sites of these five porcine AP isoforms, clearly revealing that *N*-glycosylation would potentially affect these AP isoforms’ functionality. On the other hand, accurate prediction of enzyme protein *O*-glycosylation sites is still relatively challenging [42,43].

We then investigated the role of the *N*-glycosylation in modulation of the weaned porcine intestinal AP isoform functionality through using the PNGase-F enzyme cocktail for the complete cleavage of *N*-glycosylation [36]. The recombinant PNGase-F enzyme is an amidase that only hydrolyzes the glycosyl-amine linkage between the *N*-acetyl-ß-glucosamine and the asparagine-formed *N*-glycosylation of the AP and leaves the oligosaccharide glycan moiety intact [58,67]. We conducted a dose-response experiment with the weaned porcine jejunal homogenates and observed a strong sigmoidal pattern of responses in the residual AP activity with an increasing gradient treatment doses (U/mg jejunal homogenate protein) of the PNGase F enzyme cocktail under the in vitro incubations of pH of 7.4 at 37 °C. The observed sigmoidal pattern of responses in the residual porcine jejunal AP activity was due to the complete cleavage of *N*-glycosylation by the PNGase-F enzyme cocktail; these results would suggest the fact that *N*-glycosylation of APs was allosteric regulatory intracellular biochemical events [29,68,69,70]. We subsequently showed that complete cleavage of *N*-glycosylation by the PNGase-F enzyme action considerably reduced the weaned porcine jejunal and ileal IAP isoform maximal activity without affecting the IAP enzyme affinity. Whereas complete cleavage of the *N*-glycosylation substantially decreased the cecal and colonic total AP affinity, indicating that removal of *N*-glycosylation primarily decreased the large intestinal TNAP isoform enzyme affinity without affecting the TNAP isoform activity in the weaned pig.

However, the porcine large intestinal TNAP responses to the removal of *N*-glycosylation of this study were different from a reported human intestinal cell line study in showing that TNAP activity was affected by glycosylation patterns under oxidative stress [35]. Under this context, *N*-acetyl-ß-glucosamine has been well documented in attenuating gut dysbiosis and modulating immunity and metabolic status, which is likely mediated through affecting host intestinal AP functionality [59,60,61]. Furthermore, deficiency in *O*-linked glycosylation with ß-*N*-acetylglucosamine predisposed gut to dysbiosis and inflammation in the rodent model [71]. However, potential roles of *O*-glycosylation in modulation of the AP isoforms’ functionality were not investigated in the rodent study [71], which remains to be further clarified in future research. Therefore, the in vitro cleavage of *N*-glycosylation experimental results of this study collectively suggest that *N*-glycosylation was essential to maintain the small intestinal IAP activity and the large intestinal TNAP affinity, thus differentially regulating the AP isoforms’ functionality along the weaned porcine intestinal longitudinal axis. It should be cautious that the *N*-glycosylation experimental results reported in this study are of limited in vitro nature, further in vivo studies need to be carried out to further investigate roles of *N*-glycosylation, such as dietary supplementation of glycans rich in *N*-acetyl-ß-glucosamine, in modulation of both IAP and TNAP isoform expression and functionality in the early weaned pig.

We overexpressed the porcine IAPX1 in the endotoxin-free *E. coli* strain, ClearColiBL21 (DE3) [41] and further investigated the impact of lack of glycosylation, including both *N*-linked and *O*-linked glycosylation, on the IAPX1 functional properties in this prokaryotic bacterial platform. Although the recombinant IAPX1 would, in principle, somewhat resemble to the FPLC purified weaned porcine jejunal pre-mature IAP isoforms, the recombinant IAPX1-specific affinity, with *K_m_* measured at 2102.0 ± 450.8 µM, was 6.5-fold dramatically lower than that of the FPLC purified porcine jejunal pre-mature IAP isoforms with *K_m_* averaged at 281 µM. These data further suggest that presence of glycosylation would be essential for expressing relatively high and normal porcine gut AP affinity. Furthermore, systemic differences in protein folding between the prokaryotic *E. coli* and the eukaryotic porcine species [72,73] might have also contributed to the above dramatic differences in the porcine IAP and the recombinant IAPX1 enzyme affinity *K_m_* values.

The feasibility of this recombinant porcine IAPX1 isoform overexpressed in the ClearColiBL21 (DE3) cell lysate was further investigated for its thermostability as a potential exogenous porcine IAP for potential pig nutrition, veterinary pharmaceutical as well as biochemical and molecular technological applications. Our thermostability and inhibition kinetic analyses shown that the recombinant porcine IAPX1 would lose 100% its activity after a 30 min thermal treatment at 62 °C, indicating this recombinant AP was not thermostable. These results are in agreements with the notion that mammalian AP enzymes are not thermo-tolerant [21,23,24]. High thermostability is ideally required for exogenous enzymes during the common industrial feed pelleting at high temperature (75–90 °C) for 10 min [74]. Thus, with a lack of glycosylation when expressed in the prokaryotic bacterial system, the porcine IAPX1 displayed poor functional properties particularly with limited enzyme affinity. Our results further shown that the porcine endogenous AP enzymes are not thermo-tolerant and thus may not be an ideal candidate for potentially serving as an exogenous biocatalyst.

Finally, for partitioning the porcine IAPX1 in ClearColiBL21 (DE3), we also determined the *E. coli* AP kinetics intrinsic to the ClearColiBL21 (DE3) strain under the physiological conditions. Bacterial AP and particularly *of E. coli* origins have been well characterized and reported to typically have relatively lower activity and enzyme affinity but higher thermostability [75]. In this study, the AP kinetics were not analyzed with purified enzyme protein from ClearColiBL21 (DE3), thus meaningfully comparison of the enzyme *V_max_* with literature reports was not possible. Martinez et al. [76] reported high-affinity (*K_m_* = 15–70 µM), free and secretory AP and low-affinity (*K_m_* = 0.24–1.60 mM) periplasmic-bound AP of the *E. coli* origins. Recombinant *E. coli* APs are widely used as a component in various biotechnology and molecular biology applications with a continued desire for enhancing their enzyme activity and thermostability [76]. Our thermostability results of the *E. coli* AP activity intrinsic to the ClearColiBL21 (DE3) are consistent with the literature reporting that *E. coli* AP had *TC*_50_ at 95 °C for 20 min [76]. Thus, this *E. coli* AP intrinsic to the ClearColiBL21 (DE3) is of low-affinity and low-capacity but is thermo-tolerant.

In summary, our results of this study demonstrate that *N*-glycosylation is essential for the early weaned porcine small intestinal IAP functional maturation. The further in vitro cleavage of *N*-glycosylation experimental results of this study collectively suggest that *N*-glycosylation is essential to maintain the small intestinal IAP isoform activity and the large intestinal TNAP isoform affinity, thus differentially regulating the AP isoforms’ functionality along the early weaned porcine intestinal longitudinal axis.

## 4. Materials and Methods

All chemicals were purchased from Sigma-Aldrich (St. Louis, MO, USA) unless specifically clarified.

### 4.1. Animals, Study Diets, Handling and Sample Collections

The animal utilization protocol (AUP#3370) with the following animal handling procedures was reviewed and approved by the Animal Care Committee at the University of Guelph. The pigs in this study were cared for in accordance with the guidelines established by the Canadian Council of Animal Care [77].

A total of 8 suckling barrows (Yorkshire × Landrace ♀ × Duroc ♂) at 19.3 (SE, ±0.8) d of age with an average initial body weight (BW) of 6.04 (SE, ±0.29) kg were randomly identified and weaned from different sow litters from the Arkell Swine Research Station at the University of Guelph (Arkell, ON, Canada). The weaned piglets were immediately transported into the weaned pig study room in the animal wing of the Department of Animal Biosciences at the University. Individual pigs were the experimental units; and replicate numbers for the independent in vitro enzyme kinetic experiments were further expanded through using multiple levels of the substrates for measuring target enzyme activities. The sample size and statistical power for the different experiments in this study were primarily based upon the variability (i.e., SE) of similar main endpoints reported in our relevant previous studies in weanling pigs [6].

The pigs were fed on a corn and soybean meal-based diet that was formulated to meet or exceed the nutrient requirements for the weaned piglet with BW of 6–15 kg [78] without supplementation of antibiotics or other antimicrobials in the diet (Supplemental Table S1).

All pigs had ad libitum access to the diet and water throughout the period for 22 d. At the end of the experiment, the piglets were anesthetized by inhalation of anesthetic isoflurane and sacrificed by an intra-cardiac injection of sodium pentobarbital (Schering Canada Inc., Pointe-Claire, QC, Canada) at 50 mg/kg BW [6]. The section of mesentery-free small intestinal segment to the ileo-cecal ligament was transected as the ileum [6]. The segment of the small intestine between the duodenojejunal flexure and the ileum was defined to be jejunum. Cecum was a sac-like structure between the ileo-cecal junction and the colon. Additionally, the rest of the large intestinal segment before the short rectum was colon. Tissue samples in the middle of each intestinal segments were collected and immediately rinsed with an ice-cold phosphate-buffered saline solution containing 0.100 mmol/L phenylmethylsulfonyl fluoride then flash-frozen in liquid nitrogen and stored at −80 °C. The terminal sampling for all of the piglets was completed within the morning on the same final day of the experiment. The collected porcine tissues were further finely pulverized in liquid nitrogen using a mortar and pestle for further analysis [79].

### 4.2. The AP Isoform Inhibition Experiments

The AP inhibition experiments were carried out by using the mammalian IAP-specific inhibitor of L-Phe and the TNAP-specific inhibitor of L-hArg for identifying the major AP isoforms along the four porcine intestinal segments as reported previously in the human and rodent studies [21,37,55]. The final concentrations of these two inhibitors in the reaction media mixture were 0, 10 or 100 mM, respectively. To make the 10 mM inhibitors’ final concentration, the inhibition experiments were incubated by mixing 0.050 mL weaned porcine gut tissue homogenate supernatants (2–8 mg protein content/mL) with 0.280 mL of 3.57-mM *p*NPP substrate buffer (pH at 7.4), 0.050 mL of 100-mM L-Phe or L-hArg inhibitor buffers (pH at 7.4) and 0.120 mL of a pre-*p*NPP buffer (pH at 7.4) at 37 °C for 30 min. To make the 100 mM inhibitors’ final concentration, the inhibition experiments were incubated by mixing 0.050 mL gut tissue homogenate supernatants (2–8 mg protein content/mL) with 0.020 mL of 50 mM *p*NPP substrate buffer (pH at 7.4), 0.250 mL of 200-mM L-Phe or L-hArg inhibitor buffers (pH at 7.4) and 0.180 mL of a pre-*p*NPP buffer (pH at 7.4) at 37 °C for 30 min. The pre-*p*NPP buffer contained 50 mM NaHCO_3_, 50 mM Na_2_CO_3_, 10 mM Hepes, 10 mM Tris, 6.25 mM MgCl_2_ and 2.50 mM KF at pH of 7.4 [54]. The residual AP activities measured from the 10 mM and 100 mM inhibitors’ treatments were normalized to the residual AP activities of the 0-mM inhibitor groups, i.e., the none-inhibitor baseline groups that were regarded as 100% in the 4 inhibition experiments conducted with the corresponding 4 intestinal segments.

### 4.3. The Approach-1-Purification of the Weaned Porcine Jejunal IAP Isoform by FPLC

The purification of AP isoforms was reported before [44,45,46]. Herein, 6.5-g pulverized weaned porcine jejunal tissue samples were homogenized in 100 mL of a buffer containing 20 mM Hepes buffer, 1 mM MgCl_2_ and 20 μM ZnSO_4_, 2 pieces of Pierce™ protease inhibitor tablets (Thermo Fisher Scientific, Rockford, IL, USA), 1% Triton X-100 at pH 7.4 for 3 min with a polytron homogenizer (Fisher, Pittsburgh, PA, USA), and the homogenates were then stirred at 4 °C for 1 hr. Following this, one volume of butanol (purity ≥ 99.7%) was added, and the mixture was stirred for 2 hr at 4 °C. After centrifugation at 3000× *g* for 20 min in 50-mL tubes using Beckman Coulter J6-MI centrifuge (Beckman Coulter Canada Inc., Mississauga, ON, Canada), the lower phase was resuspended and sonicated for 15 min. The precipitation by 80% ammonium sulfate was performed then desalted by passing the solution through Sephadex G-25 resin; and the target protein precipitate was further fractionated the AP protein by FPLC via loading prepared samples onto a Q-Sepharose column on an AKTA Explorer 100 system (GE Healthcare Bio-Sciences Corp., Chicago, IL, USA) and eluted with a gradient 0–1 M NaCl at pH 7.4 [47]. Eluted liquids were collected, and all fractions obtained were assayed for AP enzyme activity as described below. Purified AP fractions with relatively higher AP activity were pooled and further used in AP enzyme kinetic experiments and SDS-PAGE electrophoresis.

### 4.4. The Approach-2-Peptide N-Glycosidase F Treatment of the Porcine Intestinal Homogenates

The weaned porcine gut tissue homogenates for the PNGase-F cocktail treatment were prepared by homogenizing 0.650 g of well-pulverized tissue samples in 10 mL of an ice-cold tissue homogenization buffer at pH 7.4, containing 10 mM Hepes, 50 mM D-mannitol, 10 mM Trizma·HCl and Pierce™ protease inhibitor tablets (Thermo Fisher Scientific, Rockford, IL, USA) at a dose of 1 piece per 50 mL buffer, using a polytron homogenizer (Fisher, Pittsburgh, PA, USA). Protein contents of the tissue homogenates were measured by Bradford assay using the dye Coomassie Blue (Bio-Rad Laboratories, Hercules, CA, USA), and bovine serum albumin (Thermo Scientific, Rockford, IL, USA) was used to generate the protein standard curve [79].

The deglycosylation of AP in the weaned porcine gut mucosal homogenates was conducted with the PNGase-F kit (500 units/µL; P0704S; New England Biolabs, Ipswich, MA, USA) to remove *N*-linked oligosaccharides from glycoproteins under non-denaturing reaction conditions [36]. The PNGase-F protocol is available at link. The optimal PNGase-F dose was determined using 1–6 levels at 0, 1430, 4289, 7150, 8579, 12,868 U/mg jejunal tissue homogenate protein. The protein content of the jejunal tissue homogenate sample was 0.35 mg/mL after 20-fold dilution, representing high protein content among these tissue samples. The AP activities at the PNGase-F doses 1, 2, 3, 4 and 6 were from the slopes of the linear regression analyses in the time course experiments, whereas the AP activities of the PNGase-F of the dose 5 were from the AP kinetic experimental data by using 0.017 mmol/L *p*NPP as the chromogenic substrate. The AP activity assay conditions were further described below.

In this study, 7150 U/mg tissue protein PNGase-F was expected to be sufficient to deglycosylate *N*-glycosylation of glycoproteins in the tested gut tissue samples. Then tissue homogenates and 2 µL of GlycoBuffer 2 (10×) were combined to make a 20-µL total reaction volume; PNGase-F was added, content mixed gently and incubated at 37 °C overnight. After incubation, the AP kinetics in tissue homogenates of the four intestinal segments were determined. For the no enzyme groups, gut homogenate samples were incubated under the same conditions in the absence of the PNGase-F.

### 4.5. The Approach-3-Overexpression of the Recombinant Porcine IAPX1 Isoform in the ClearColi BL21 (DE3)

While it is commonly believed that bacteria have limited or rare *O*- and *N*-glycosylation systems, Schäffer and Messner’s review found that certain species of bacteria do possess post-translational *N*-glycosylation modifications to their expressed glycoproteins [40,80]. Forsberg et al. [36] reported that a phytase of bacterial origin was not *N*-glycosylated when expressed in *E. coli*; however, this bacterial phytase was heavily *N*-glycosylated when expressed in pigs. Thus, *E. coli* was chosen as the protein expression host to further examine the roles of *O*- and *N*-glycosylation in the porcine IAP isoform functionality. The overexpression of the porcine IAP isoform in an endotoxin-free *E. coli* strain, referred to as the ClearColiBL21 (DE3) [41], would also potentially facilitate commercial application of the porcine endogenous IAP as an exogenous AP in commercial pig nutrition and veterinary pharmaceutical applications. The porcine IAP isoform gene X1, i.e., the IAPX1 (protein product ID: XP_003133777.1-X1), was commercially synthesized by Integrated DNA Technologies as a gene block (Coralville, IA, USA). The gene block of IAPX1 isoform was then ligated into the vector p15TV-L (accession number EF456736) and fused in frame with an N-terminal His-tag for generating p15TV-L-IAPX1. This plasmid construct was transformed into the ClearColiBL21 (DE3) using standard protocols with similar procedures reported in our previous studies [81]. The construct was verified by DNA sequencing in the Guelph Molecular Super Center.

Both the ClearColiBL21 (DE3) harboring p15TV-L-IAPX1 and p15TV-L alone were inoculated separately into 1 L Luria-Bertani (LB) medium (Thermo Fisher Scientific, Rockford, IL) with 100 μg/mL ampicillin and incubated with shaking at 37 °C overnight. Then, isopropyl-β-D-thiogalactopyranoside was added at final concentration of 0.5 mmol/L when optical density of medium at 600 nm met 0.8 and continued the culture with shaking at 15 °C overnight in the New Brunswic Innova-44 incubator (Eppendorf Canada Ltd., Mississauga, ON, Canada). The cells were harvested by centrifugation at 5000× *g* and 4 °C for 15 min and cell pellets were resuspended in 15 mL a cell lysis buffer containing 300 mM NaCl, 10 mM imidazole and 50 mM sodium HEPES at pH 7.0 [81]. The resuspension was further disrupted by sonication (Fisher, Pittsburgh, PA, USA) and lysed cell homogenates, referred to as cell lysates, were immediately stored at −80 °C for the further protein purification efforts described below and/or for the direct use in the AP kinetics and thermostability assays.

To purify the overexpressed IAPX1 by following our previously established procedures for another similar *E. coli* BL21 (DE3) cell system [81], the frozen lysed cell homogenates were thawed and the cell debris were removed by centrifugation at 17,500× *g* for 10 min. The supernatants were filtered through a 0.45-µm filter and mixed with the Ni-NTA resin for 40 min with the gentle stirring under 4 °C. The mixtures were poured into a column and the resin in a column for affinity chromatography and were washed with 300 mL of a buffer containing 300 mM NaCl, 50 mM HEPES and 20 mM imidazole at pH 7.0. The protein product of IAPX1 were eluted with the same buffer but containing higher 250 mM imidazole. Likely due to the buried fusing N-terminal His-tag, we experienced difficulties in purifying the anticipated IAPX1 protein via the Ni-NTA resin column system that we had used previously [81]. Thus, the recombinant IAPX1 was not further characterized with its purified protein.

### 4.6. Enzyme Activity Assay and Kinetic Experiments

The AP activities were measured by incubating samples in the presence of magnesium using different levels of *p*NPP as the artificial chromogenic substrate at 37 °C and pH 7.4 for 30 min in an assay buffer containing 50 mM NaHCO_3_, 50 mM Na_2_CO_3_, 10 mM Hepes, 10 mM Tris, 5 mM MgCl_2_ and 2.08 mM KF in four replicates. Reactions were terminated by adding 0.50 mL of 0.50 M NaOH before reading the absorbance of produced *p*-nitrophenol (*p*NP) at 400 nm using BioTek Synergy H1 microplate reader (BioTek, Winooski, VT, USA). One AP enzyme activity unit (U) is defined to be = 1 nmol/min. The AP activity is expressed as nmol/(mg protein·min) or U/mg protein.

Specifically, the AP activity assay was performed using 3.125 mM *p*NPP as a substrate in 0.50 mL of an assay buffer at pH 7.4 and 37 °C for 30 min. In time-course experiments, samples were incubated with 0.03 mM *p*NPP in 0.50 mL of an assay buffer at pH 7.4 and 37 °C for 0, 15, 30, 45 min. The AP kinetics of the processed porcine gut tissue samples were assayed by incubating samples using *p*NPP with 10 gradient concentrations ranging 0–0.8 mmol/L in incubation media at pH 7.4 and 37 °C for 30 min.

To mathematically partition the recombinant IAPX1-specific enzyme activity overexpressed in the ClearColiBL21 (DE3) cell lysate, kinetic experiments for the total AP activity were determined with the ClearColiBL21 (DE3) cell lysates processed from the same condition-cultured cells harboring the p15TV-L-IAPX1 and overexpressing IAPX1 by using the *p*NPP with 16 gradient concentrations ranging 0–6 mM in incubation media at pH 7.4 and 37 °C for 30 min. Then, kinetic experiments for the AP activity only intrinsic to the *E. coli* (ClearColi BL21 (DE3)) cell were carried out with the ClearColiBL21 (DE3) cell lysates processed from the same condition-cultured cells but only harboring the vector p15TV-L alone by using the *p*NPP with 16 gradient concentrations ranging 0–6 mM in incubation media at pH 7.4 and 37 °C for 30 min.

### 4.7. Thermostability of the Porcine Recombinant AP Isoform IAPX1

To mathematically partition the thermostability responses for the recombinant IAPX1-specific residual AP enzyme activity overexpressed in the ClearColiBL21 (DE3) cell lysate, thermostability experiments for the total AP activity were conducted with the ClearColiBL21 (DE3) cell lysates processed from the same condition-cultured cells harboring the p15TV-L-IAPX1and overexpressing IAPX1 and further subjecting to the gradient thermal treatments, ranging from the room temperature 22 to 80 °C in a heating block for 30 min. Then, kinetic experiments for the AP activity only intrinsic to the *E. coli* (ClearColiBL21 (DE3)) cell were carried out with the ClearColiBL21 (DE3) cell lysates processed from the same condition-cultured cells but only harboring the vector p15TV-L alone and further subjecting to the gradient thermal treatments, ranging from the room temperature 22 to 80 °C in the heating block for 30 min. Residual AP activities associated with the above thermal treated cell lysates were measured by using 20 mM *p*NPP as a substrate in 0.50 mL of the assay buffer at pH 7.4 and 37 °C for 30 min as described before.

### 4.8. SDS-PAGE for the Porcine Jejunal IAP Isoform Fractions

After being isolated using FPLC, the protein fractions containing weaned porcine jejunal IAP were denatured by heating them at 100 °C for 5 min with the addition of 2-mercaptoethanol. The resulting denatured proteins were loaded onto Mini-PROTEAN TGX Stain-Free Precast Gels (Bio-Rad Laboratories, Hercules, CA, USA) in 20 µL solutions containing 0.39–4.27 µg of protein. The jejunal IAP protein bands were separated using the SDS-PAGE electrophoresis and photographed with stain-free imaging system (Bio-Rad Laboratories, Hercules, CA, USA). The molecular weights of the target IAP protein bands were estimated using Image Lab (v5.0, Bio-Rad Laboratories, Hercules, CA, USA).

### 4.9. Identification of AP Isoform Genes in the Porcine Genome (Sscrofa11.1) and the Prediction of the AP Protein Post-Translational N-Glycosylation Modifications

The genomic mining of the porcine intestinal AP isoform genes was based on sequences from the porcine genome database (*Sus scrofa* Ver. 11.1). Four porcine AP isoform genes were identified, including 3 porcine IAP isoform genes located in the chromosome 15; and 1 porcine TNAP gene but expressing two distinct TNAP isoforms in the chromosome 6, resulting in a total of 5 porcine AP isoforms that are all likely expressed along the porcine small and the large intestinal longitudinal axis. Glycosylphosphatidylinositol-anchoring site was predicted on GPI-SOM (http://gpi.unibe.ch/ (accessed on 23 March 2020)). Signal peptide motif was predicted by the online servers of Signal IP 5.0 (http://www.cbs.dtu.dk/services/SignalP/ (accessed on 23 March 2020)) and coding amino acid (AA)-based molecular weights (MW) for the 5 porcine AP isoforms were calculated on ExPASy (https://web.expasy.org/compute_pi/ (accessed on 23 March 2020)).

### 4.10. Modeling of Porcine AP Isoforms’ N-Glycosylation Sites and Architecture of Catalytic Sites

*N*-Glycosylation site prediction was conducted on NetNGly (http://www.cbs.dtu.dk/services/NetNGlyc/ (accessed on 23 March 2020)). Specifically, the porcine IAP isoforms and the TNAP isoforms are highly conserved in AA sequences, respectively, but with various glycosylation patterns, which will further contribute to differences in these porcine AP isoform molecular weights primarily expressed on the enterocyte apical membrane. The three-dimensional models mapped with the predicted *N*-glycosylation sites of the 5 porcine AP isoforms and their architectures of catalytic sites were generated by the SWISS-MODEL online server using the crystal structure of a homologous human placenta AP (RCSB-PDB#:1EW2) as a template [82]. Structure images were generated using PyMOL (www.pymol.org (accessed on 23 March 2020)).

### 4.11. Kinetic Calculations and Statistical Analyses

Plotting of AP enzyme activity (Y, nmol/(mg protein·min)) against the PNGase-F doses (x, U/mg protein respective gut tissue sample) was best fitted by a sigmoidal model, as described in Equation (1).

(1) $$Y=\mathrm{Bo}+\left(\mathrm{To}-\mathrm{Bo}\right)/[1+10^{\left(\mathrm{ED}50-x\right)*\mathrm{Hill}\;\mathrm{Slope}}]$$

where Bo is the Y value at the bottom plateau; To is the Y value at the top plateau; ED50 (50% effective dose) is the value when the reaction is halfway between Bo and To; and the Hill Slope illustrates the curve steepness.

The AP kinetics of the *E. coli* (ClearColiBL21 (DE3)) cell lysate and the partitioned recombinant IAPX1-specific enzyme activity overexpressed in the same cell lysate were determined by fitting a standard Michaelis–Menten model. The plotting of inhibition kinetic relationships between the residual AP activity intrinsic to the *E. coli* (ClearColiBL21 (DE3)) cell lysate and the partitioned recombinant IAPX1-specific enzyme activity overexpressed in the ClearColiBL21 (DE3) cell lysate in responses to the increasing gradient of external thermal treatments was respectively fitted according to a standard quadratic regression model and the simple natural exponential regression model according to Equation (2).

(2) $$Y=J_{0}e^{\mathrm{bx}}$$

where Y is the residual AP activity (J, nmol/(mg protein·min; or % of the control)) in the cell lysates in responses to the respective external thermal treatments at x (I, °C); J_0_ is the initial residual AP activity (nmol/(mg protein·min; or % of the control)), i.e., the initial residual AP activity at the starting external thermal treatment temperature; and the constant b is called the rate constant or rate of the residual AP activity change as affected by the respective external thermal treatments.

The thermostability kinetic parameter estimates were further analyzed by using the Eadie–Hofstee linear model shown in Equations (3) and (4) [79,83].

(3) $$J=I_{MIN}-TC_{50}*\left(J/I\right)$$

(4) $$I_{MAX}=I_{C}-I_{MIN}$$

where J is residual AP activity intrinsic to the *E. coli* (ClearColiBL21 (DE3)) cell lysate and the partitioned recombinant IAPX1-specific enzyme activity overexpressed in the ClearColiBL21 (DE3) cell lysate (nmol P-nitrophenol/(mg protein·min) and as relative residual activity (as % of the positive control group measured at the room temperature at 22 °C)); *I_MIN_* is the minimal inhibited AP activity intrinsic to the *E. coli* (ClearColiBL21 (DE3)) cell lysate and the partitioned recombinant IAPX1-specific enzyme activity overexpressed in the ClearColiBL21 (DE3) cell lysate (nmol *p*-nitrophenol/(mg protein·min) and as relative residual activity (as % of the positive control group measured at the room temperature at 22 °C)]; *TC*_50_ is temperature in Celsius at half of the maximal inhibited AP activity (*I_MAX_*) intrinsic to the *E. coli* (ClearColiBL21 (DE3)) cell lysate and the partitioned recombinant IAPX1-specific enzyme activity overexpressed in the ClearColiBL21 (DE3) cell lysate by the external thermal treatment; *I_MAX_* is the maximal inhibited AP activity intrinsic to the *E. coli* (ClearColiBL21 (DE3)) cell lysate and the partitioned recombinant IAPX1-specific enzyme activity overexpressed in the ClearColiBL21 (DE3) cell lysate (nmol *p*-nitrophenol/(mg protein·min) and as relative residual activity (as % of the positive control group measured at the room temperature at 22 °C)]; and *I_C_* is the positive control group mean AP activity intrinsic to the *E. coli* (ClearColiBL21 (DE3)) cell lysate and the partitioned recombinant IAPX1-specific enzyme activity overexpressed in the ClearColiBL21 (DE3) cell lysate (nmol p-nitrophenol/(mg protein·min) and as relative residual activity (as % of the positive control group measured at the room temperature at 22 °C)].

The sigmoidal, Michaelis-Menten, Eadie–Hofstee simple linear, quadratic and the simple exponential plotting were all performed by using Graphpad Prism (v8.3.0, GraphPad Software Inc., San Diego, CA, USA). Significance (*p* < 0.05) of some kinetic parameter estimates was examined by Tukey’s tests. Some enzyme kinetic parameter estimates were also compared between and/or among treatments by the pooled two-tailed Student’s *t*-test [84].

Analyses of variances (ANOVA) of the endpoints were compared by the GLIMMIX procedure. Multiple comparisons among the target porcine gut segments were carried out by Tukey’s tests. All of these statistical analysis procedures, including the ANOVA, Tukey’s tests and the above sigmoidal, Michaelis–Menten, Eadie–Hofstee simple linear, quadratic and the simple exponential equation analyses were conducted by using SAS (v9.4; SAS Institute Inc., Cary, NC, USA). *p* values < 0.05 were considered significant differences of comparisons; and *p* values < 0.10 indicated a trend of significant differences of comparisons.

## Figures and Tables

**Table 1:** Summary of the porcine genomic survey-based signal peptide motifs, glycosylphosphatidylinositol (GPI)-anchoring sites, *N*-glycosylation sites and the predicted non-glycosylated pre-mature coding AA-based molecular weights (MW) of various porcine alkaline phosphatase (AP) isoforms.

Isoform ^1^	NCBI Protein Accession Code	Predicted Signal Peptide Motif	Predicted GPI-Anchoring Site	*N*-Glycosylation Sites	Pre-Mature AP MW ^2^
IAPX1	XP_003133773.1	1-19	C-30	N141	52.7 kDa
IAPX2	XP_020930975.1	1-19	C-31	N141	52.8 kDa
IAPX3	XP_003133777.1	1-19	C-31	N141, N421	52.8 kDa
TNAPX1	XP_020953340.1	No	C-26	N279, N320, N352, N479	60.8 kDa
TNAPX2	XP_020953343.1	1-17	C-26	N230, N271, N303, N430	53.3 kDa

^1^ The porcine intestinal-type alkaline phosphatase (IAP) isoform-X1, -X2 and -X3 of IAPX1, IAPX2 and IAPX3; and the tissue-nonspecific alkaline phosphatase (TNAP) isoform-X1 and X2 of TNAPX1 and TNAPX2. ^2^ The predicted pre-mature molecular weights of the AP isoforms are estimated based on their coding AA sequences without including their saccharide moieties, their signal peptides and their GPI anchor regions.

**Table 2:** In the Approach-1 research, comparisons of kinetics of activity of hydrolyzing the chromogenic substrate *p*-nitrophenyl phosphate by the chromatographically separated and purified intestinal-type isoforms of alkaline phosphatase (IAP) fractions from the weanling porcine jejunal tissue homogenates.

IAP Fraction ^1^	*K_m_*, µmol/L	*V_max_*, nmol/(mg protein·min)
Fraction-1-IAP	22.28 ± 5.32 ^a^	89.26 ± 3.98 ^a^
Fraction-2-IAP	53.12 ± 8.47 ^b^	56.75 ± 2.28 ^b^
Fraction-3-IAP	327.0 ± 27.20 ^c^	349.1 ± 13.00 ^c^
Fraction-4-IAP	233.7 ± 21.30 ^d^	131.2 ± 4.80 ^d^

Values are parameter estimates ± SE, n = 30, representing kinetic parameter estimates obtained from one kinetic experiment using mean measurements of 3 replicates at each substrate level and 10 different substrate media concentrations. ^a,b,c,d^ Different superscript letters within the same column indicate significant differences, *p* < 0.05. The kinetic parameter estimates were compared by the pooled two-tailed Student’s *t*-test. ^1^ Fraction-1-IAP, glycosylated and partially glycosylated mature IAP mixture; Fraction-2-IAP, predominantly glycosylated and mature IAP; Fraction-3-IAP, minor glycosylated mature and major non-glycosylated pre-mature IAP mixture; and Fraction-4-IAP, minor glycosylated mature and primarily non-glycosylated pre-mature IAP mixture, as further visualized in Figure 2.

**Table 3:** In the Approach-2 research, effects of removal of *N*-glycosylation through in vitro incubations with the peptide *N*-glycosidase F (PNGase F) cocktail along the intestinal longitudinal axis of jejunum, ileum, cecum and colon in the weanling pigs.

Item	Untreated ^1^	No Enzyme	PNGase F	SEM ^2^	*p* Values
Jejunum					
*K_m_*, µmol/L	25.47	29.55	29.21	4.75	0.7985
*V_max_*, nmol/(mg protein·min)	27.74 ^a^	43.26 ^b^	12.05 ^c^	4.04	0.0001
Ileum					
*K_m_*, µmol/L	24.67	32.96	20.59	3.95	0.1035
*V_max_*, nmol/(mg protein·min)	21.26 ^a^	39.30 ^b^	11.73 ^ac^	2.81	<0.0001
Cecum					
*K_m_*, µmol/L	22.08	45.85	182.30	50.81	0.0787
*V_max_*, nmol/(mg protein·min)	3.08	5.54	3.99	0.82	0.1248
Colon					
*K_m_*, µmol/L	39.50 ^ab^	23.58 ^a^	59.51 ^b^	6.88	0.0054
*V_max_*, nmol/(mg protein·min)	10.00	15.50	11.04	2.52	0.2841

^1^ Untreated group: no overnight incubation; No enzyme group: after an incubation in the base buffer alone without the PNGase F enzyme cocktail but subjecting to intrinsic effects of the respective gut tissue homogenates; PNGase F group: after an incubation in the enzyme-buffer mixture containing 7150 U PNGase F/mg protein from the respective gut tissue homogenates. ^2^ Kinetic parameter estimate means ± pooled SEM (n = 8), representing kinetic parameter estimate means obtained from eight kinetic experiments with eight individual pig gut tissue sample homogenates; and each kinetic experiment conducted with measurements of 4 replicates at each substrate level and 10 different substrate media concentrations, as shown in Figure 6. ^a,b,c^ Different superscript letters in the same row indicate significant differences, *p* < 0.05, as compared by using Tukey’s test of SAS.

**Table 4:** In the Approach-3 research, partitioning of activity kinetics of the porcine intestine-type alkaline phosphatase (IAP) isoform-X1 (IAPX1) recombinant overexpressed in the *E. coli* (ClearColiBL21 (DE3)) cell lysate in hydrolyzing the chromogenic substrate *p*-nitrophenyl phosphate.

Item	*K_m_*, µmol/L	*V_max_*, nmol/(mg protein·min)
IAPX1-overexpressed *E. coli* cell lysate ^1^	3064.0 ± 339.5 ^a^	1.64 ± 0.07 ^a^
The *E. coli* cell lysate without the IAPX1 overexpression ^2^	4068.0 ± 525.0 ^b^	0.89 ± 0.05 ^b^
The IAPX1-specific kinetics partitioned ^3^	2102.0 ± 450.8 ^c^	0.75 ± 0.05 ^c^

Values are parameter estimates ± SE, n = 64, representing kinetic parameter estimates obtained from one respective kinetic experiment using measurements in 4 replicates at each substrate level and 16 different substrate media concentrations shown in Figure 7. ^1^ The kinetics of the total AP activity determined with the *E. coli* (ClearColiBL21 (DE3)) cell lysates processed from the same condition-cultured cells harboring the p15TV-L-IAPXI and overexpressing IAPXI. ^2^ The kinetics of the AP activity intrinsic to the *E. coli* (ClearColiBL21 (DE3)) cell lysates processed from the same condition-cultured cells but only harboring the vector p15TV-L alone. ^3^ The IAPX1-specific kinetics of the AP activity partitioned between the total AP activity determined with the *E. coli* (ClearColiBL21 (DE3)) cell lysates overexpressing the IAPXI and the AP activity only intrinsic to the *E. coli* (ClearColiBL21 (DE3)) cell lysates processed from the same condition-cultured cells but only harboring the vector p15TV-L alone. ^a,b,c^ Different superscript letters within the same column indicate significant differences, *p* < 0.05, as compared by the pooled two-tailed Student’s *t*-test.

**Table 5:** In the Approach-3 research, comparative summary of the in vitro enzyme thermostability kinetics.

J ^1^	Thermostability Kinetic Parameter Estimates			
	*TC*_50_ ^2^	*I_MIN_* ^3^	*I_MAX_* ^4^	*I_C_* ^5^
Residual AP activity, nmol/(mg protein·min)				
The *E. coli* alone	NS ^6^	NS	NS	1.16 ± 0.03
The IAPX1-specific	31.12 ± 3.70	0 ^7^	1.20 ± 0.08	1.36 ± 0.08
Relative enzyme activity, % of the corresponding control				
The *E. coli* alone	NS	NS	NS	100.00 ± 2.59
IAPX1-specific	31.11 ± 3.71	0 ^8^	100.00 ± 5.58	100.00 ± 5.58

Values are parameter estimates ± SE (n = 24) obtained through the Eadie–Hofstee linear analyses, representing the thermostability kinetic parameter estimates obtained from six inhibition experiments using measurements in four replicates within each of the experiments showing in Figure 8 and Figure 9. ^1^ J, residual AP activity (nmol/(mg protein·min)) determined by using the *E. coli* (ClearColiBL21 (DE3)) cell lysates; and residual AP activity (nmol/(mg protein·min)) for the IAPX1-specific and partitioned between the total AP activities determined with the ClearColiBL21 (DE3) cell lysates overexpressing IAPXI and the AP activities only intrinsic to the ClearColiBL21 (DE3) cell lysates; as well as relative residual AP activity for the ClearColiBL21 (DE3) cell alone and the IAPX1-specific partitioned, as % of the positive control group measured, at room temperature at 22 °C. ^2^ *TC*_50_, temperature in Celsius (°C) at half of the maximal inhibited intrinsic *E. coli* (ClearColiBL21 (DE3)) cellular AP activity and the partitioned IAPX1-specific activity (*I_MAX_*) by the external thermal treatments according to Equation (3). ^3^ *I_MIN_*, the minimal residual intrinsic *E. coli* (ClearColiBL21 (DE3)) AP activity and the partitioned IAPX1-specific AP activity (nmol/(mg protein·min)); and as % of the positive control group measured at the room temperature at 22 °C according to Equation (3). ^4^ *I_MAX_*, the maximal inhibition of the intrinsic *E. coli* (ClearColiBL21 (DE3)) AP activity and the partitioned IAPX1-specific activity (nmol/(mg protein·min)); and as % of the positive control group measured at the room temperature at 22 °C according to Equation (4). ^5^ *I_C_*, average intrinsic *E. coli* (ClearColiBL21 (DE3)) AP activity and the partitioned IAPX1-specific activity (nmol/(mg protein·min)); and as % of the positive control group measured at the room temperature at 22 °C. ^6^ NS, not significant parameter estimates, indicating these parameter estimates could not be obtained from significant Eadie–Hofstee linear analyses (*p* > 0.05 for the linear term slope) as shown in Figure 8B,D. ^7^ *I_MIN_* estimated for the IAPX1-specific AP residual activity at 0.1526 (±0.1354) nmol/(mg protein·min) was not different from zero (*p* = 0.2718) as compared by the pooled two-tailed Student’s *t*-test and as shown in Figure 9B. ^8^ *I_MIN_* estimated for the relative IAPX1-specific AP residual activity at 11.27 (±9.99) as % of the corresponding control *I_C_* was not different from zero (*p* = 0.2718), as compared by the pooled two-tailed Student’s *t*-test and shown in Figure 9D.

## Data Availability

All final essential data, including graphs and images, generated or analyzed during this study are included in and/or supplied as Supplemental Materials to this published article. The raw datasets as well as intermediate calculations and statistically analyzed data during the current study are available from the corresponding author on reasonable request.

## Supplementary Materials

The following are available online at https://www.mdpi.com/article/10.3390/pathogens12030407/s1, Table S1: Formulation of the experimental weanling pig diet.

## Abbreviations

AA	amino acids
AP	alkaline phosphatases
BW	body weight
FPLC	fast protein-liquid chromatography
L-hArg	L-homoarginine
IAP	intestinal-type AP
*Ic*	mean enzyme activity of control group
*I_MAX_*	maximal magnitude of inhibition in the enzyme activity
*I_MIN_*	minimal residual enzyme activity
*K_m_*	enzyme catalytic affinity
LPS	lipopolysaccharides
MW	molecular weight
PAMPs	pathogen-associated-molecular patterns
L-Phe	L-phenylalanine
PNGase F	peptide *N*-glycosidase F
*p*NP	*p*-nitrophenol
*p*NPP	*p*-nitrophenyl phosphate
*TC*_50_	temperature in Celsius (°C) at half of the maximal inhibited AP activity
TNAP	tissue-nonspecific AP
*V_max_*	maximal enzyme activity
