# Supplementary material for: Deglycosylation Differentially Regulates Weaned Porcine Gut Alkaline Phosphatase Isoform Functionality along the Longitudinal Axis
Source: Pathogens. 2023 Mar 3;12(3):407. doi: 10.3390/pathogens12030407 (PMC10053101; doi:10.3390/pathogens12030407)
Supplement: Supplementary file 1 [file pathogens-12-00407-s001.zip › pathogens-2182816-supplementary.pdf]

pathogens
MDPI

Supplemental Materials

Table S1: Formulation of the experimental weanling pig diet (on as-fed basis).

Item	g/kg diet
Corn	513.8
Cornstarch[1]	3.2
Soybean meal	320.0
Dried whey powder	120.0
Animal fat-oil blend	5.2
Lysine-HCl (79%)	2.8
DL-Methionine (purity, 99%)	1.5
L-Threonine (99%)	1.0
Monocalcium phosphate	10.5
Limestone ($CaCO_3$)[2]	11.2
Iodized salt	2.5
Vitamin-Mineral premix[3]	5.0
Sweetener	0.5
Titanium oxide[5]	3.0
Digestible energy, DE, [6] kcal/g	3.4
Crude protein, CP, [7] %	19.6
Total dietary fiber, [6] TDF, %	12.7
Neutral detergent fiber, NDF, [6] %	7.8
Acid detergent fiber, ADF, [6] %	3.6
Hemicellulose, [6] %	4.2
Total soluble fiber, [6] %	4.8

Total calcium, Ca, [6] %	0.8
Total phosphorus, P, [6] %	0.7
Total Ca/total P ratio[8]	1.2
Total sodium, Na, [6] %	0.2
Total chlorine, Cl, [6] %	0.4

[1]Conventional corn starch commercially available.
[2]Included the amounts of limestone ($CaCO_3$) contributed from the DSM (Cambridge, ON, Canada)
[3] Swine vitamin-mineral premix from the DSM supplied the following vitamins (IU, µg, mg or g/kg diet) and trace minerals (mg/kg diet) in the diets (as-fed basis): vitamin A (Rovimix A 1000 and Rovimix AD3 1000/200), 10000 IU; vitamin E (Rovimix E 50 Adsorbate US), 40 IU; vitamin D (Rovimix AD3 1000/200), 1000 IU; vitamin K_3 (menadione sodium bisulfite, MSBC), 2.5 mg; pantothenic acid (Calpan Feed Xinfa), 15 mg; thiamine (vitamin B_1 feed grade), 1.50 mg; folic acid (Rovimix Folic 80 50), 2.0 mg; riboflavin (Rovimix B2 80 SD Totes), 5 mg; pyridoxine (vitamin B_6 as Pyridoxine HCl), 1.50 mg; vitamin B_{12} (1% feed grade), 25 µg; biotin (Rovimix Biotin HP), 0.20 mg; niacin (Rovimix niacinamide), 25 mg; choline (choline chloride, 70%), 0.50 g; Cu (copper sulfate, purity 25%), 15 mg; Fe (ferrous sulfate, purity 30%), 100 mg; Mn (manganese sulfate, purity 31.5%), 20 mg; Se (sodium selenite, purity 4.0%),0.30 mg; Zn (zinc sulfate, purity 35.5%), 105 mg; and I (potassium iodide), 0.50 mg.
[4]Aureomycin provides 550 mg of chlortetracycline hydrochloride per kg diet and purchased from the Floradale Feed Mill (Floradale, ON, Canada) with a veterinary prescription at the University of Guelph.
[5]Purchased from Fisher Scientific, Fair Lawn, NJ.
[6]Calulated values (NRC 1998; 2012).
[7]Analyzed values.
[8]Standardized digestible P content calculated from the NRC (2012).
